# A1AT dysregulation of metabolically stressed hepatocytes by Kupffer cells drives MASH and fibrosis

**DOI:** 10.1038/s12276-025-01408-1

**Published:** 2025-02-12

**Authors:** Jeong-Su Park, Jin Lee, Feng Wang, Hwan Ma, Zixiong Zhou, Yong-Sun Lee, Kwangyeon Oh, Haram Lee, Guoyan Sui, Sangkyu Lee, Yoon Mee Yang, Jang-Won Lee, Yong-Ha Ji, Chun-Woong Park, Hwan-Soo Yoo, Bang-Yeon Hwang, Sang-Bae Han, Nan Song, Soohwan Oh, Bumseok Kim, Ekihiro Seki, Jin Tae Hong, Yoon Seok Roh

**Affiliations:** 1 College of Pharmacy and Medical Research Center, Chungbuk National University, Cheongju, South Korea; 2 Department of Pathology, School of Medicine, University of California, San Diego, CA USA; 3 Department of Pathology and Institute of Oncology, The School of Basic Medical Sciences, Fujian Medical University, Fuzhou, China; 4 Toxicological Evaluation and Research Department, National Institute of Food and Drug Safety Evaluation, Ministry of Food and Drug Safety, Cheongju, South Korea; 5 College of Pharmacy, Korea University, Sejong, South Korea; 6 College of Pharmacy, Sungkyunkwan University, Suwon, South Korea; 7 College of Pharmacy, Kangwon National University, Chuncheon, South Korea; 8 Research and Development Center, MediTake Co. Ltd., Suwon, South Korea; 9 College of Veterinary Medicine and Biosafety Research Institute, Jeonbuk National University, Iksan, South Korea; 10 Karsh Division of Gastroenterology and Hepatology, Cedars–Sinai Medical Center, Los Angeles, CA USA; 11 Samuel Oschin Comprehensive Cancer Institute, Cedars–Sinai Medical Center, Los Angeles, CA USA

**Keywords:** Chronic inflammation, Extracellular signalling molecules

## Abstract

Metabolic dysfunction-associated steatohepatitis (MASH) is associated with the activation of Kupffer cells (KCs) and hepatic stellate cells, at which point a metabolically stressed hepatocyte becomes integral to the progression of the disease. We observed a significant reduction in the level of alpha-1-antitrypsin (A1AT), a hepatocyte-derived secreted factor, in both patients with MASH and mice fed a fast-food diet (FFD). KC-mediated hepatic inflammation, most notably IL-1β, led to the transcriptional inhibition of A1AT by HNF4α. In quintuple *Serpina1a–e* knockout mice, ablation of A1AT worsened MASH through increased activity of proteinase 3 (PR3), a proinflammatory protease produced by F4/80^hi^/CD11b^low^/TIM4^−^/CCR2^+^ monocyte-derived KCs (MoKCs). Conversely, A1AT restoration or PR3 inhibition mitigated MASH progression. A PR3-bound cytokine array identified IL-32 as a key factor associated with MASH. Combining *IL-32* with *SERPINA1*, the gene encoding A1AT, synergistically predicted patients at risk of MASH through univariate logistic regression analysis. Furthermore, in vivo overexpression of IL-32γ alleviated MASH induced by FFD. However, additional knockout of A1AT increased PR3 activity, consequently abolishing the anti-MASH effects of IL-32γ. Blocking PR3-mediated IL-32γ cleavage via the V104A mutation sustained its protective actions, while the PR3-cleaved C-terminal fragment activated KCs. Additionally, after cleavage, the antifibrogenic effect of IL-32γ is lost, resulting in a failure to prevent the activation of hepatic stellate cells. This study highlights the critical role of hepatocyte-derived A1AT in the PR3/IL-32γ axis during MASH development. Strategies to correct A1AT dysregulation, such as A1AT supplementation or PR3 inhibition with sivelestat, may offer protection against the development and progression of MASH and fibrosis.

**Figure:**
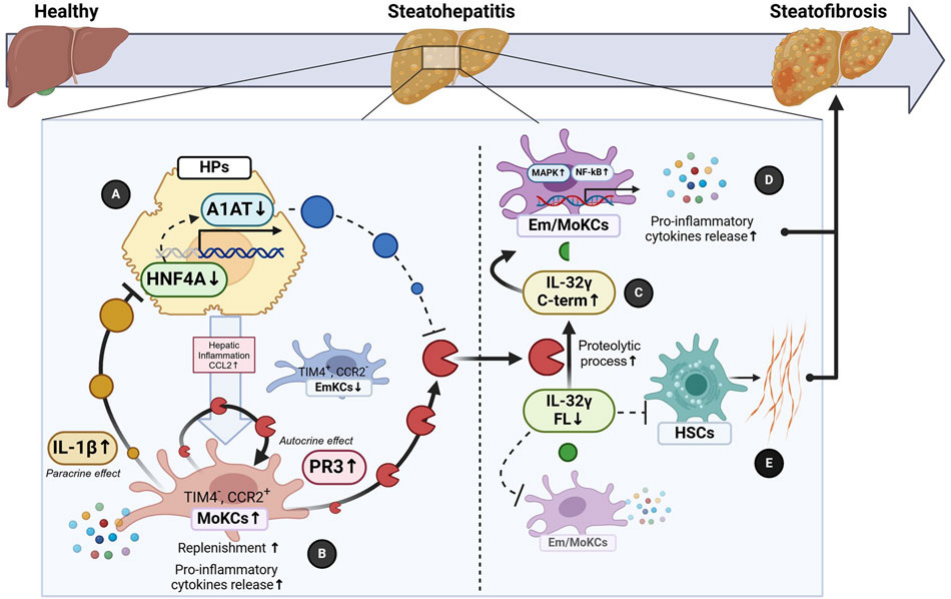
Elevated hepatic IL-1β levels in MASH lead to the downregulation of A1AT via the transcription factor HNF4α, resulting in increased recruitment of proinflammatory MoKCs and heightened PR3 activity. PR3 cleaves IL-32γ, transforming it from an anti-inflammatory and antifibrogenic cytokine into a potent activator of KCs and failing to prevent HSC activation. This cascade amplifies liver inflammation and fibrosis, suggesting that targeting the A1AT/PR3/IL-32γ axis could be a strategy for treating MASH.

Elevated hepatic IL-1β levels in MASH lead to the downregulation of A1AT via the transcription factor HNF4α, resulting in increased recruitment of proinflammatory MoKCs and heightened PR3 activity. PR3 cleaves IL-32γ, transforming it from an anti-inflammatory and antifibrogenic cytokine into a potent activator of KCs and failing to prevent HSC activation. This cascade amplifies liver inflammation and fibrosis, suggesting that targeting the A1AT/PR3/IL-32γ axis could be a strategy for treating MASH.

## A1AT deficiency drives inflammation in metabolic liver disease

Metabolic dysfunction-associated steatotic liver disease (MASLD) is a major cause of liver failure worldwide. Researchers are trying to understand how it progresses to more severe conditions such as metabolic dysfunction-associated steatohepatitis (MASH). This study focuses on a protein called alpha-1-antitrypsin, which is important for liver health. The researchers used mice and human samples to study the role of A1AT in liver disease. They found that A1AT levels are lower in people and mice with MASLD, which leads to increased inflammation and liver damage. They also discovered that a protein called proteinase 3 becomes more active when A1AT is low, worsening the condition. By experimenting with mice, they showed that increasing A1AT or blocking PR3 can reduce liver damage. This suggests new treatment possibilities for MASH. The study concludes that targeting the A1AT/PR3 pathway could help manage liver disease progression.

This summary was initially drafted using artificial intelligence, then revised and fact-checked by the author.

## Introduction

Metabolic dysfunction-associated steatotic liver disease (MASLD)^1^ is increasingly recognized as a leading cause of liver failure worldwide. The increasing prevalence, progression and serious consequences of MASLD have escalated it to become a global health priority^2,3^. Severe hepatocellular damage, lipotoxicity and hepatic inflammation, which are primarily regulated by Kupffer cells (KCs), along with the excessive accumulation of extracellular matrix proteins synthesized by hepatic stellate cells (HSCs), substantially increase the risk of developing metabolic dysfunction-associated steatohepatitis (MASH) and advanced fibrosis^4^. However, despite extensive research efforts, the complex molecular mechanisms and intrahepatic cell interactions driving the progression from MASLD to clinically significant MASH and fibrosis have not been fully elucidated. These findings underscore the need for detailed research to identify the key molecular regulators driving the progression of MASH, ultimately aiming to develop enhanced therapeutic targets.

Central to our study is alpha-1-antitrypsin (A1AT), an acute-phase glycoprotein predominantly produced by hepatocytes and known as a serine protease inhibitor^5^. A1AT deficiency, a genetic disorder, is known to cause liver and lung damage due to the accumulation of mutant A1AT and the dysregulation of neutrophil elastase^6^. However, the implications of A1AT deficiency, especially in nonhereditary contexts within MASH, remain poorly understood. In MASH, there is an influx of immune cells and a notable increase in the number of monocyte-derived Kupffer cells (MoKCs), resulting from the replacement of embryo-derived Kupffer cells (EmKCs) with MoKCs, which leads to an amplified inflammatory response^1,7,8^. The proteins secreted from these cells, particularly proteinase 3 (PR3), play crucial roles in immune responses, contributing to the proteolytic processing of proinflammatory cytokines and receptors, as well as microbial clearance^9,10^. Previous studies have established that neutrophils are the primary source of PR3 secretion^11,12^. Notably, monocyte-derived PR3 has been implicated in exacerbating conditions such as cystic fibrosis and small vessel vasculitis^13,14^. Understanding how dysregulated proteolytic enzymes influence disease progression and regulate cytokine processing could pave the way for the development of MASH treatments. However, specific mechanisms supporting this hypothesis in MASH remain largely unexplored.

IL-32, a key modulator in various inflammatory diseases, including asthma, neuronal diseases, metabolic disorders and cancers, has nine identified isoforms^15,16^. Among these, IL-32γ is reported to be the most active and plays a crucial role in immune modulation^17–19^. Recent studies have correlated IL-32 with non-alcoholic fatty liver disease activity score (NAS), insulin resistance and aminotransferase levels, suggesting a close association with MASLD^20,21^. However, the detailed mechanistic role of IL-32γ in MASLD/MASH pathogenesis remains elusive.

Our study focused on the relationships among intrahepatic cells, particularly major liver cell types such as hepatocytes, KCs and HSCs, in the context of MASH. Through secretome profiling of primary hepatocytes from fast-food diet (FFD)-fed mice, we identified A1AT-5, a paralog of A1AT, as the most downregulated protein, which has been corroborated in human subjects and various MASLD mouse models. The genetic deletion of A1AT resulted in increased replenishment of MoKCs, which contributed to the increased activity of PR3. PR3 not only induced inflammation in KCs but also altered the biological activity of IL-32γ through proteolytic degradation and cleavage, affecting both KCs and HSCs in humans and mice. These findings reveal a critical mechanism of MASH progression involving A1AT/PR3-mediated proteolytic processing of IL-32γ, which regulates KC and HSC activity. Moreover, our study reveals potential targeted therapeutic avenues for MASH and its related conditions, offering a viable therapeutic strategy.

## Materials and methods

### Human and mouse serum analysis

The human serum samples from 56 patients with MASLD and related data used for this study were provided by the Biobank of Ajou University Hospital, Keimyung University Hospital, Gyeongsang National University Hospital, Pusan National University Hospital, Seoul National University Hospital, Jeju National University Hospital and Chungbuk National University Hospital (members of the Korea Biobank Network; ethical code: CBNU-201906-BR-0118 and CBNU-202210-BR-0223). Serum samples from patients with MASLD and healthy controls were analyzed via Human Serpina1 DuoSet enzyme-linked immunosorbent assay (ELISA) (R&D system, DY1268), human neutrophil elastase/ELA2 DuoSet ELISA (R&D system, DY9167-05) and Human Proteinase 3/PRTN3 DuoSet ELISA (R&D system, DY6134-05), according to the manufacturer’s manuals. Mouse blood samples were collected via cardiac puncture, and the serum was separated via centrifugation (500 rpm for 15 min). The levels of alanine transaminase (ALT), aspartate aminotransferase (AST) and cholesterol were analyzed via Green Cross Labcell and Raonbio. Mouse serum samples were analyzed via a Mouse Alpha-1-Antitrypsin ELISA kit (Icllab, E-90A1T), a Mouse Neutrophil Elastase/ELA2 DuoSet ELISA kit (R&D Systems, DY4517-05) and a Mouse PR3 ELISA kit (Abcam, ab277720), according to the manufacturers’ instructions.

### Mouse models

Wild-type (WT) (C57BL/6NJ) mice were obtained from Samtako Bio. A1AT knockout (KO)/NJ (C57BL/6J-Serpina1em3Chmu/J, 035015) mice were obtained from Jackson Laboratory. The generation of hIL-32γ Tg mice has been previously described^22^. Two strains (C57BL/6J-Serpina1em3Chmu/J and hIL-32g Tg mice) were bred to generate hIL-32γ/A1AT KO mice. The animals were fed an FFD (40% calories from fat, 0.2% cholesterol, RD Western Diet, OpenSource Diets, plus 23.1 g/l fructose and 18.9 g/l glucose added to the drinking water)^23–25^ or a normal chow diet (NCD) for 24 weeks. The animals were provided free access to food and water for 24:weeks. The mice were treated with either Respreeza (2 mg/kg, twice a week, intraperitoneal injection) or sivelestat (50 mg/kg, three times a week, intraperitoneal injection) during the last 6 weeks of the feeding protocol. All the mice were viable and fertile and had no overt phenotypes, considering their tissues or organs. All animal experiments and experimental protocols were performed according to the guidelines of the Chungbuk National University Institutional Animal Care and Use Committee (ethical code CBNUA-2233-24-02). The animals were maintained in a specific pathogen-free facility under controlled conditions of temperature (21 ± 2 °C), humidity (50 ± 10%), lighting (12 h artificial light and dark cycles) and air exchange. High pressure was maintained in the experimental room to prevent contamination.

### Histological analysis of the murine liver

Liver tissues were collected from the mice, fixed with formalin and embedded in paraffin. The paraffinized slides were deparaffinized and rehydrated for hematoxylin and eosin (H&E) staining, immunohistochemistry (IHC), and Sirius red staining. To visualize liver structure and immune cell infiltration, liver sections were stained with H&E (Leica Biosystems, 3801698). To visualize collagen deposition, liver sections were stained with Sirius red solution (Sigma-Aldrich, 365548). Before IHC staining, the antigens were retrieved via a target retrieval solution (Invitrogen, 2085704). The sections were blocked with a ready-to-use blocker (Vector Lab, ZF0506) and incubated with the primary antibody PR3 (Santa Cruz, sc-74534) overnight at 4 °C. For the detection of IHC staining, we utilized a mouse-on-mouse immunodetection kit (Vector Lab, BMK-2202). For BODIPY staining, liver tissues were fixed overnight in formalin, hydrated in 10% sucrose for 3 h and then placed in 30% sucrose for another 24 h. The liver tissue was then embedded in Tissue-Tek optimal cutting temperature compound (Sakura, C4583) for frozen tissue sectioning. The fluorescent compound BODIPY (Thermo Fisher Scientific, D3922) was used to visualize lipids in the tissue sample. For quantitative analysis of lipid and fibrosis areas, whole scanned liver sections were captured via a DMi8 Leica microscope at 200× magnification, and positive areas were measured via LAS X (Leica) and ImageJ software (National Institutes of Health).

### Secretome proteomics

To screen for unidentified hepatokines via proteomic analysis, primary hepatocytes from the NCD- and FFD-fed mice were isolated and seeded with EX-CELL 325 protein-free CHO serum-free medium (Sigma-Aldrich, 14340C). The conditioned media (CM) was collected after 24 h and centrifuged at 3000*g* to remove floating cells and cellular debris. The supernatants were concentrated by ultrafiltration using 10K molecular weight cutoff protein concentrators (Thermo Fisher Scientific) for proteomics analysis. In brief, the collected protein was reduced with 200 mM Tris (2-carboxyethyl) phosphine and alkylated with 60 mM iodoacetamide. After the protein was precipitated via chilled acetone, the pellets were digested with sequencing grade modified trypsin (Promega) at 37 °C for 16 h. The digested peptides were labeled with a six-plex tandem mass tag isobaric mass tagging kit (Thermo Fisher Scientific) and fractionated with a high-pH reversed-phase peptide fractionation kit (Thermo Fisher Scientific) according to the manufacturer’s protocol. After the desalting process, each sample was analyzed via nanoliquid chromatography-electrospray ionization tandem mass spectrometry (MS/MS). The resulting MS/MS data were processed via MaxQuant with the integrated Andromeda search engine (v.1.5.1.0). MS/MS spectra were searched against the UniProt Human database from UniProt (http://UniProt.org). The false discovery rate thresholds for protein, peptide and modification sites were specified at 1%.

### Protein purification

Plasmids of the N- and C-terminal domains of IL-32γ cleaved by PR3 were transferred to a pPROEX/HTa expression vector via EcoRI and XabI restriction enzyme sites. Protein purification of each IL-32γ domain was performed by Bionics Co. and Celantec via the open reading frame of IL-32γ. Briefly, BL21 (DE3) was used as the *Escherichia coli* strain and cultured in media (100 ml of LB + 100 µg/ml ampicillin) overnight. For cell culture scale-up, *E.* *coli* was cultured in 1 l of media with 1 mM IPTG induction at 37 °C for 4 h. The *E.* *coli* pellet was then generated by centrifugation at 4500 rpm for 10 min and stored at −70 °C. For lysis, the cell pellet was resuspended completely in His-binding buffer (20 mM sodium phosphate dibasic, 500 mM NaCl and 20 mM imidazole, pH 7.4). Then, 50 mg/ml lysozyme (final concentration of 1 mg/ml) and 100 mM phenylmethylsulfonyl fluoride (final concentration of 1 mM) were added to the cell pellet. The samples were subsequently centrifuged at 20,000*g* for 30 min at 4 °C. The ÄKTA Go protein purification system was used for His affinity purification according to the manufacturer’s manual.

### Human and mouse hepatic transcriptomes, RNA-seq and scRNA-seq

Human RNA-sequencing (RNA-seq) data were analyzed via publicly available Gene Expression Omnibus (GEO) datasets, including GSE48452, GSE61260 and GSE135251. Single-cell RNA-seq (scRNA-seq) data were obtained from the Single Cell Portal hosted by the Broad Institute, specifically from the dataset titled ‘Identification of a Broadly Fibrogenic Macrophage Subset Induced by Type 3 Inflammation: Human Liver Fibrosis scRNA-seq Atlas’, available at https://singlecell.broadinstitute.org. scRNA-seq data from various publicly accessible studies (GSE185477, GSE125188, GSE156625, GSE192740, GSE115469 and GSE136103) were consolidated. The RNA-seq data generated for IL-1β-stimulated hepatocytes in this study have been deposited in the GEO repository and are accessible under the accession number GSE282137.

### Statistical analysis

Statistical analyses were performed via an unpaired Student’s *t*-test with GraphPad Prism 7 (GraphPad Software Inc.). Statistical significance was set at *P* < 0.05. All the data are reported as the means ± s.e.m.

Other materials, reagents and protocols are described in the supplementary material and methods.

## Results

### A1AT, a hepatokine, is downregulated in humans and mice with MASLD

**Fig. 1:**
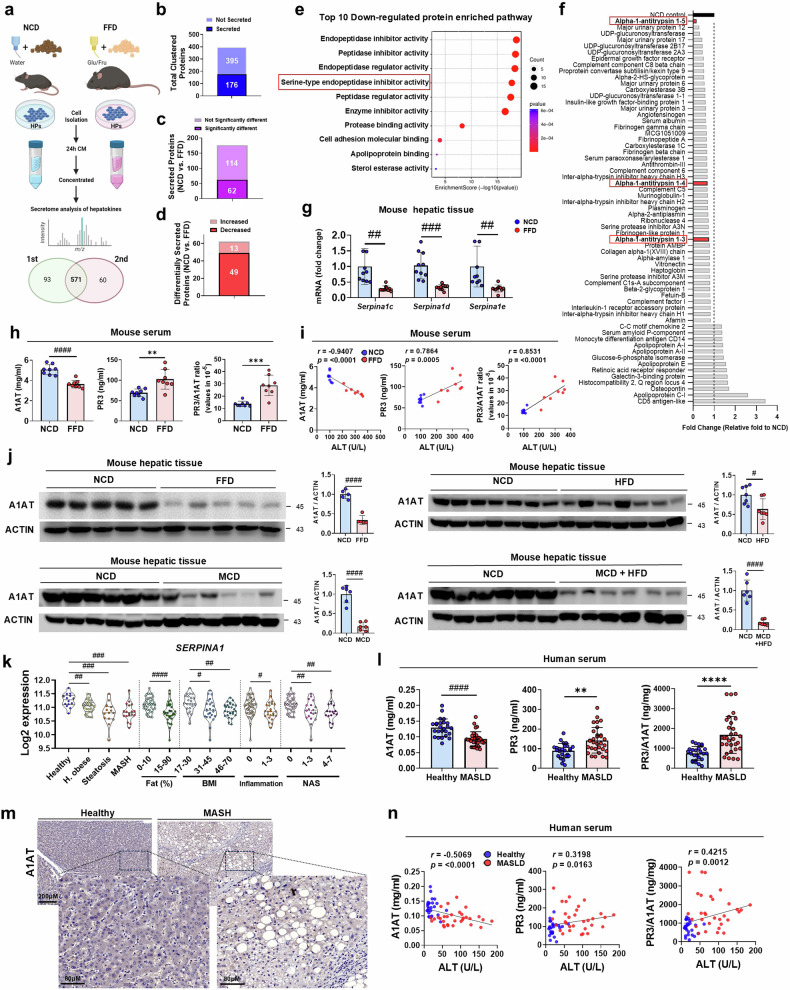
Downregulation of A1AT under MASLD/MASH conditions exacerbates hepatic inflammation and fibrosis. **a**‒**d**, Two replicative secretome proteomics analyses of CM from NCD- and FFD-fed (24-week-old) mice indicating total clustered proteins (**a**), secreted proteins (**b**), significantly different proteins (**c**) and differentially secreted proteins (**d**). **e**, Enrichment analysis of downregulated proteins revealed in the secretome proteomics study. **f**, The list of proteins from the secretome proteomics analysis showing the relative fold change compared with NCD–HCM. **g**, Relative hepatic tissue expression of *Serpina1c-e* mRNA in the NCD- and FFD-fed mice. **h**, Serum levels of A1AT detected in the NCD- and FFD-fed mice via ELISA. **i**, Correlations of A1AT, PR3 and the PR3/A1AT ratio with the serum ALT level in the NCD- and FFD-fed mice. **j**, Protein expression of A1AT in the hepatic tissue of preclinical models, such as the FFD (24 week feeding), HFD (24 week feeding), MCD (4 week feeding) and MCD–HFD (6 week feeding)-induced MASLD models. **k**, Human gene expression of A1AT in accordance with different disease states, fat composition, BMI, inflammation score and NAS. **l**, Detection of the serum levels of A1AT and PR3 and the PR3/A1AT ratio in the control group (healthy) and patients with MASLD via ELISA. **m**, Detection of A1AT protein expression in control groups (healthy) and patients with MASH via IHC staining. **n**, Correlations of A1AT, PR3 and the PR3/A1AT ratio with the serum ALT and AST levels in the control group (healthy) and patients with MASLD. The data are presented as the means ± s.d., *n* ≥ 5; *^,#^*P* < 0.05, **^,##^*P* < 0.01, ^***,###^*P* < 0.001 and ^****,####^*P* < 0.0001 versus the control model. ns, not significant.

To elucidate the interaction mechanism between hepatocytes and KCs in MASLD, we first cultured primary KCs with hepatocyte-conditioned medium (HCM) from mice fed either a NCD or an FFD. Compared with those treated with HCM–NCD, KCs treated with HCM from FFD-fed mice (HCM–FFD) presented upregulated proinflammatory gene expression (Supplementary Fig. 1a). These findings suggest that hepatokines from FFD-fed mice create an environment conducive to inflammatory responses, thus activating KCs. Next, we conducted secretome profiling of HCM from mice fed either an NCD or FFD (Fig. 1a). Using an algorithm that identifies secreted proteins with an N-terminal signal sequence^26^, we detected 176 secreted proteins out of a total of 571 proteins (Fig. 1b). Among these proteins, 62 were significantly different between the NCD and FFD groups (Fig. 1c), with 13 upregulated and 49 downregulated in the FFD group compared with the NCD group (Fig. 1d). The results of the protein‒protein interaction analysis revealed three interconnected clusters, with two major clusters highlighting enzyme regulatory activity (Supplementary Fig. 1b). The enrichment analysis^27^ of the upregulated proteins revealed associations with diabetes and MASLD-related pathways, including cholesterol metabolism, fat digestion and absorption, complement and coagulation cascades, and lipids and atherosclerosis (Supplementary Fig. 1c). These pathways are known to play critical roles in regulating lipid metabolism and hepatic inflammation.

Furthermore, enrichment analysis of the significantly downregulated secreted proteins revealed impairments in enzyme regulator activity, serine-type endopeptidase inhibitor activity and proteolysis regulation (Fig. 1e). Notably, secretome profiling revealed that alpha-1-antitrypsin 1-5 (A1AT-5) was the most significantly downregulated protein (Fig. 1f). Bulk gene expression analysis of human tissues confirmed that A1AT (*SERPINA1*) is predominantly expressed in the liver (Supplementary Fig. 1d), with single-cell expression data indicating that *SERPINA1* is primarily localized in hepatocytes in both humans and mice (Supplementary Fig. 1e, f). Consistent with these findings, hepatic mRNA expression of three murine A1AT paralogs (*Serpina1c-e*), identified via secretome proteomics analysis, was significantly downregulated in FFD-fed mice (Fig. 1g). Additionally, FFD-fed mice presented a notable reduction in serum A1AT levels, accompanied by an increase in the levels of PR3, a serine protease primarily inhibited by A1AT, and an elevated PR3/A1AT ratio (Fig. 1h). These alterations were significantly correlated with increased ALT and cholesterol levels (Fig. 1i and Supplementary Fig. 2a). The downregulation of hepatic mRNA and protein expression of A1AT was further validated in various preclinical models, including the FFD, high-fat diet (HFD), methionine- and choline-deficient diet (MCD), MCD–HFD, choline-deficient amino acid-defined high-fat diet (CDA–HFD), diabetic obese and liver disease progression aggravation diet-induced MASH models (Fig. 1j and Supplementary Fig. 2b-e).

In line with our proteomic and transcriptomic data from mice, human hepatic A1AT gene expression was significantly downregulated in association with disease state, fat composition, body mass index (BMI), hepatic inflammation score and NAS (Fig. 1k). Consistent with those in the FFD-induced MASH model, the serum levels and tissue expression of A1AT were notably decreased, whereas the PR3 levels and the PR3/A1AT ratio were increased in the serum samples from patients with MASLD/MASH (Fig. 1l, m). Interestingly, the levels of neutrophil elastase, another A1AT target with higher selectivity than PR3 (ref.^28^), remained unchanged (Supplementary Fig. 2f). A1AT, PR3 and the PR3/A1AT ratio were significantly correlated with ALT and AST levels (Fig. 1n and Supplementary Fig. 2g). These findings collectively highlight that downregulated A1AT and upregulated PR3 are associated with the progression of MASLD/MASH conditions in both humans and mice.

### A1AT deletion exacerbates hepatic inflammation and fibrosis in mice

**Fig. 2:**
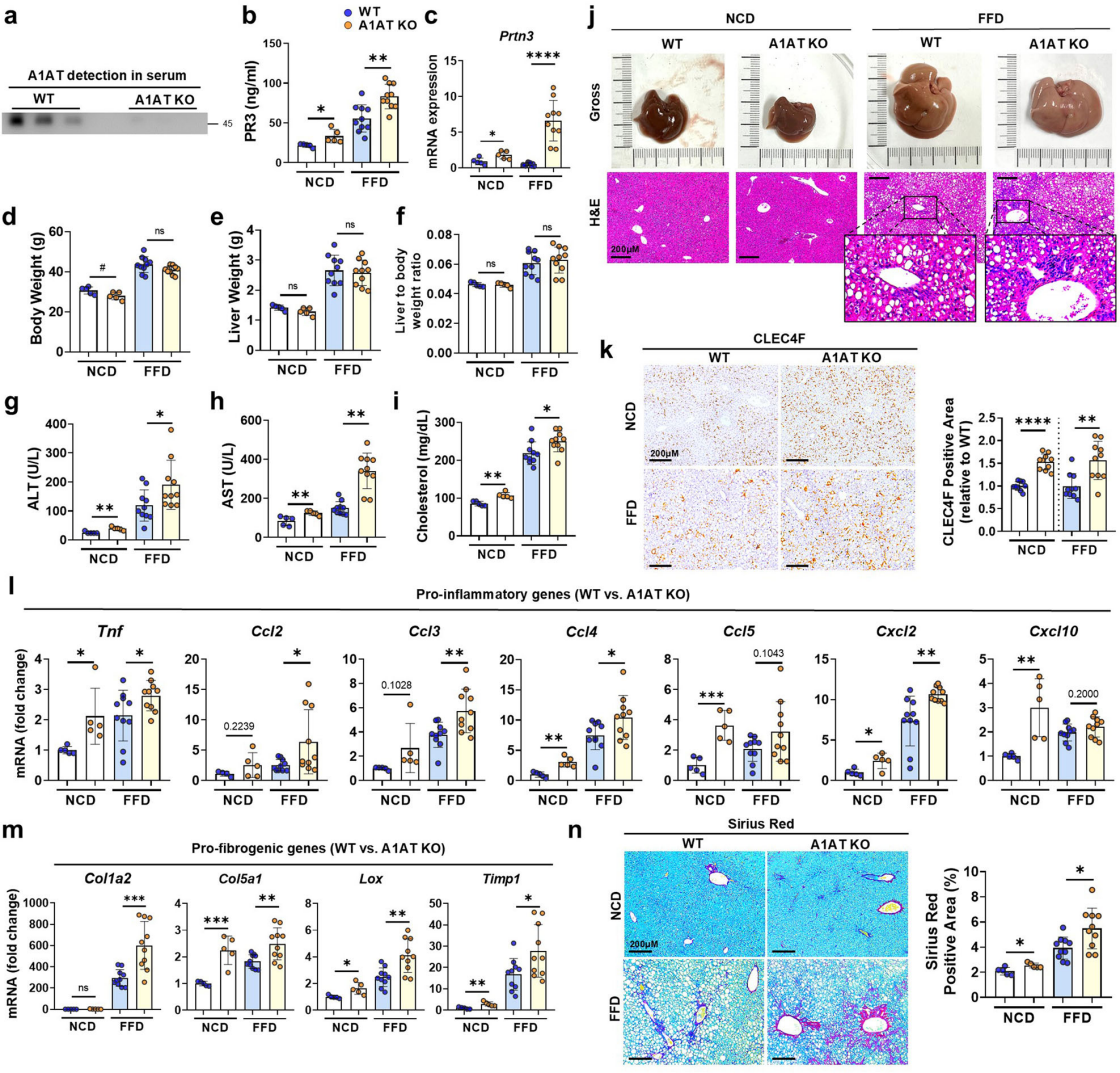
Quintuple deletion of *Serpina1a–e* exacerbates MASH progression with increased hepatic inflammation and fibrosis. **a**, Serum A1AT protein levels in A1AT KO and WT mice. **b**, Serum PR3 levels in NCD-fed WT (*n* = 5), NCD-fed A1AT KO (*n* = 5), FFD-fed WT (*n* = 10) and FFD-fed A1AT KO (*n* = 10) mice. **c**, Hepatic tissue mRNA expression of *Prtn3* in each group. **d**–**i**, BW (**d**), LW (**e**), LBW ratio (**f**) and serum levels of ALT (**g**), AST (**h**) and cholesterol (**i**) in each group. **j**, Representative images of freshly collected liver tissues and H&E-stained liver sections from each group. **k**, Representative immunohistochemical staining of CLEC4F-positive KCs in liver tissue sections from each group. Quantification of the CLEC4F-positive area relative to that of WT control mice in both the NCD and FFD groups. **l**, Relative mRNA expression of proinflammatory genes in hepatic tissues from each group. **m**, Relative mRNA expression of profibrogenic genes in hepatic tissues from each group. **n**, Representative images of Sirius red-stained liver tissue sections from each group. The data are presented as the means ± s.d., *n* ≥ 5; *^,#^*P* < 0.05, **^,##^*P* < 0.01; ***^,###^*P* < 0.001 and ****^,####^*P* < 0.0001 versus the control model. ns, not significant.

To assess whether A1AT depletion serves as a critical mediator in the pathogenesis of MASH, we utilized quintuple *Serpina1a–e* KO (A1AT KO) mice fed either an NCD or an FFD. We first validated A1AT KO by confirming the absence of serum A1AT expression (Fig. 2a). Additionally, we observed a significant increase in the PR3 level in both the serum and hepatic tissue (Fig. 2b, c). While NCD-fed A1AT KO mice presented a significant reduction in body weight (BW) compared with that of NCD-fed WT mice, there were no differences in liver weight (LW) or the body-to-liver weight ratio between A1AT KO and WT mice after FFD feeding (Fig. 2d–f). However, A1AT KO mice presented higher levels of ALT, AST and cholesterol than WT mice did (Fig. 2g–i). Histological analysis revealed increased immune cell infiltration in FFD-fed A1AT KO mice, along with a notable increase in the CLEC4F-positive KC population in both NCD- and FFD-fed A1AT KO mice (Fig. 2j, k). Consistent with these findings, the expression levels of genes associated with hepatic inflammation (*Tnf*, *Ccl2*, *Ccl3*, *Ccl4*, *Ccl5*, *Cxcl2* and *Cxcl10*) and fibrosis (*Col1a2*, *Col1a5*, *Lox* and *Timp1*), as well as collagen deposition, were significantly increased in A1AT KO mice (Fig. 2l–n). These findings demonstrate that the absence of A1AT significantly increases serum PR3 levels, contributing to FFD-induced liver inflammation and fibrosis.

### Modulation of the A1AT/PR3 axis through A1AT restoration and PR3 inhibition effectively blocks MASH progression in mice

**Fig. 3:**
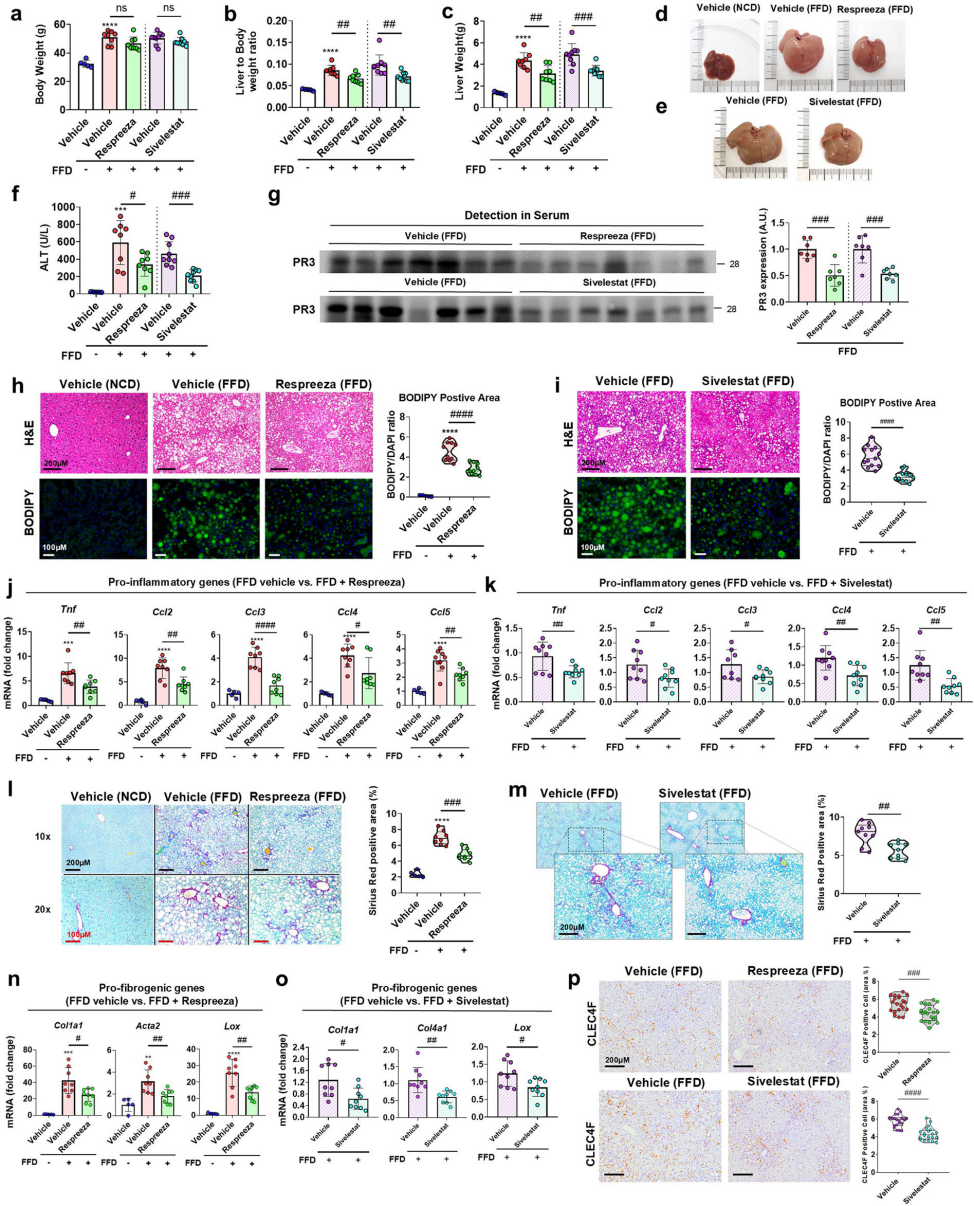
A1AT supplementation or PR3 inhibition improves steatohepatitis and fibrosis in mice. **a**–**f,** BW (**a**), LW (**b**), LBW ratio (**c**), appearance of freshly collected liver tissue samples: treated with Respreeza (**d**) and sivelestat (**e**), and serum levels of alanine transferase (**f**) in the vehicle (NCD), vehicle (FFD), Respreeza (FFD) and sivelestat (FFD) groups. NCD-fed vehicle, *n* = 5; FFD-fed vehicle, *n* = 8 and FFD-fed and Respreeza-treated groups, *n* = 8. FFD-fed vehicle-fed, *n* = 10 and FFD-fed and sivelestat-treated groups, *n* = 10. **g**, Serum PR3 levels in each group using immunoblot. **h**,**i**, H&E- and BODIPY-stained liver sections. Each representative image of a BODIPY-stained section was selected and measured to determine the BODIPY-positive area (green, for lipid accumulation) and the 4,6-diamidino-2-phenylindole (DAPI)-positive area (blue, for nuclei and normalization). **j**, Relative mRNA expression of proinflammatory genes in hepatic tissues from the vehicle (NCD), vehicle (FFD) and Respreeza (FFD) groups. **k**, Relative mRNA expression of proinflammatory genes in hepatic tissues from the vehicle (FFD) and sivelestat (FFD) groups. **l**,**m**, Sirius red-stained liver sections from each group. Each representative image of a whole-body liver section from each group was analyzed to determine the Sirius red-positive area. **n**,**o**, Relative expression of profibrogenic genes in hepatic tissue from each group. **p**, IHC staining image of CLEC4F-positive KCs in each group. The data are presented as the means ± s.d; *^,#^*P* < 0.05, **^,##^*P* < 0.01, ***^,###^*P* < 0.001 and ****^,####^*P* < 0.0001 versus the control model. ns, not significant.

We identified the dysregulated A1AT/PR3 axis as a key mediator of hepatic inflammation during MASH progression. To explore the therapeutic potential of targeting this axis, we evaluated the effects of Respreeza, a human serum-derived A1AT, and sivelestat, a PR3 inhibitor, in a mouse model. In vivo treatment with both Respreeza and sivelestat did not significantly affect the BW of the mice, but effectively mitigated the diet-induced increases in LW, the liver-to-body weight (LBW) ratio and the levels of ALT and cholesterol (Fig. 3a–f and Supplementary Fig. 3a). Additionally, the inhibition of PR3 activity was confirmed in the serum of both the Respreeza- and sivelestat-treated groups (Fig. 3g). Histological analyses revealed that both the Respreeza and sivelestat treatments reduced hepatocellular ballooning degeneration and lipid accumulation (Fig. 3h, i). In these drug-treated groups, a significant decrease in the hepatic expression of proinflammatory genes and the production of hepatic TNF was observed (Fig. 3j, k and Supplementary Fig. 3b–d). Furthermore, liver fibrosis deposition was ameliorated, as indicated by reduced Sirius red-positive areas and decreased mRNA expression of profibrogenic genes (Fig. 3l–o). Notably, a decrease in CLEC4F-positive KCs was observed in the groups treated with Respreeza and sivelestat (Fig. 3p). Collectively, these results suggest that the combined pharmacological approach of A1AT supplementation and PR3 inhibition offers therapeutic potential for treating steatohepatitis and fibrosis by modulating the A1AT/PR3 balance in MASH.

### IL-1β suppresses A1AT expression via HNF4α in hepatocytes

**Fig. 4:**
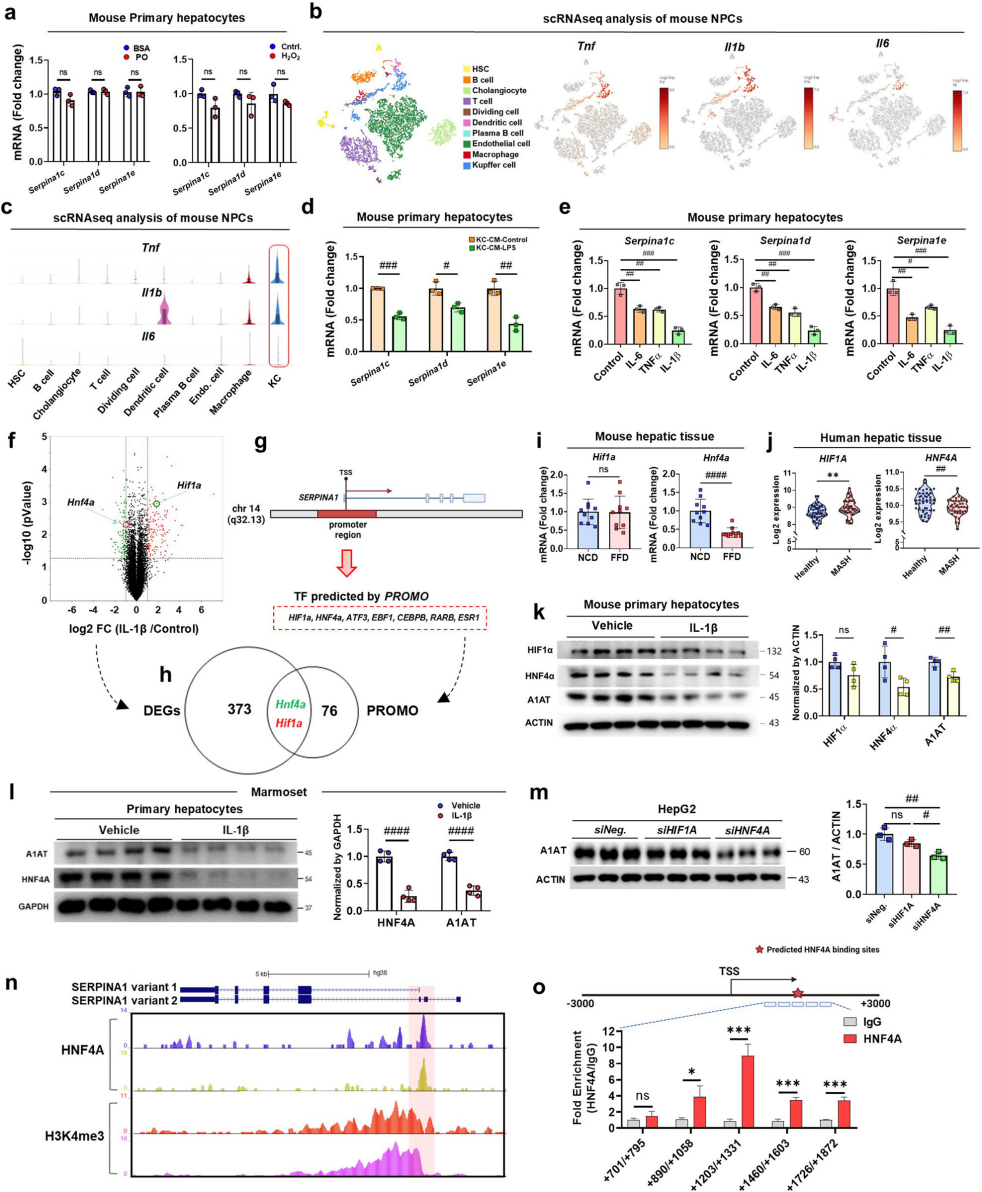
Elevated IL-1β leads to HNF4α-mediated suppression of A1AT expression in hepatocytes. **a**, Relative mRNA expression of *Serpina1c-e* in hepatocytes treated with palmitic acid, oleic acid (PO) and hydrogen peroxide (H_2_O_2_). **b**, scRNA-seq analysis of NPCs from the NCD- and FFD-fed mice. Visualization of the scRNA-seq data highlighting the gene expression patterns of the cytokines *Tnf*, *Il1b* and *Il6*. **c**, A violin plot visualization of the scRNA-seq data, highlighting the gene expression patterns of the cytokines *Tnf*, *Il1b* and *Il6* in different cell types. **d**, Relative expression of *Serpina1c-e* mRNA in hepatocytes treated with CM from LPS-simulated (CM-KC-LPS) or untreated KCs (CM-KC-Control). **e**, Relative expression of *Serpina1c-e* mRNA in primary hepatocytes treated with recombinant TNF, IL-6 and IL-1β proteins. All recombinant proteins were treated at 100 ng/ml, for 24 h. **f**, A volcano plot of differential mRNA expression between the IL-1β recombinant protein-treated group and the untreated group (control). The selected genes with *P* values (*P* < 0.05) and fold changes (>1.5) are labeled with green/red dots reflecting down-/upregulated genes. **g**, Prediction of *SERPINA1* gene promoter-binding transcription factors via PROMO software (version 3.0.2) with 3,000 bp upstream to 3000 bp downstream of the *SERPINA1* transcription initiation site (TSS) located in the promoter region. **h**, A Venn diagram indicating the integration of the differentially expressed genes (DEGs) (**f**) and prediction of transcription factors (**g**). **i**, Relative mRNA expression of *Serpina1* gene transcription factor candidates (*Hif1a* and *Hnf4a*) in hepatic tissue samples from NCD- and FFD-fed mice. **j**, mRNA expression levels of *HIF1A* and *HNF4A* in human hepatic tissues were analyzed via publicly available human liver RNA-seq datasets in the GEO database (GSE48452 and GSE61260). **k**, Protein levels of HIF1α, HNF4α and A1AT in hepatocytes treated with recombinant IL-1β protein or untreated hepatocytes (control). **l**, Primary hepatocytes isolated from marmosets were stimulated with IL-1β for 48 h. Protein expression of A1AT and HNF4α was detected. **m**, Relative protein expression of A1AT in HepG2 cells transfected with siHIF1a, siHNF4a or the siNegative control (siNeg.). **n**, Browser track showing HNF4A (from GSM469863 and GSM469864) and H3K4me4 (GSM2534178 and GSM2534179) ChIP-seq data in the *SERPINA1* region. Pink highlights depict the *SERPINA1* variant 1 promoter. **o**, The binding affinity of HNF4α for the *SERPINA1* promoter in HepG2 cells was investigated via a ChIP assay. This involved the use of an HNF4α antibody and an IgG control. Additionally, RT‒PCR experiments were conducted with various primer combinations to further examine this interaction. The data are presented as the means ± s.d.; *^,#^*P* < 0.05, **^,##^*P* < 0.01, ***^,###^*P* < 0.001 and ****^,####^*P* < 0.0001 versus the control model. ns, not significant.

We next focused on the transcriptional regulation of A1AT in the context of MASH. Initially, we challenged hepatocytes with palmitic acid and H_2_O_2_, which are stimuli associated with the unfolded protein response (UPR) and endoplasmic reticulum (ER) stress in MASLD^29^. However, these stimuli did not result in the downregulation of A1AT mRNA expression (Fig. 4a). Given the notable elevation of proinflammatory cytokines in both FFD-fed mice and patients with MASH^30^, we identified KCs as the primary cytokine source via scRNA-seq of nonparenchymal cells (NPCs), and these findings were further confirmed by in vivo and in vitro experiments (Fig. 4b, c and Supplementary Fig. 4a, b). Treatment of hepatocytes with conditioned media from lipopolysaccharide (LPS)-stimulated KCs led to a marked reduction in A1AT mRNA expression (Fig. 4d). Furthermore, direct treatment of primary hepatocytes with recombinant proinflammatory proteins (TNF, IL-6 and IL-1β) significantly downregulated *Serpina1c-e* expression (Fig. 4e). To elucidate the molecular mechanism underlying *Serpina1* downregulation, we conducted RNA-seq analysis of IL-1β-stimulated hepatocytes. Using a digital gene expression analysis (Fig. 4f) and the prediction of *Serpina1* promoter-binding transcription factors (Fig. 4g), we identified two candidates, *Hnf4a* and *Hif1a*, following intersection analysis (Fig. 4h). Hepatic mRNA expression levels of *HNF4A* and *HIF1A* were examined in both mice and human subjects, revealing significant downregulation of *HNF4A* in both FFD-fed mice and patients with MASH (Fig. 4i, j). This decrease in *HNF4A* expression was also confirmed via single-cell expression analysis of human hepatocytes from patients with low and mild steatosis (Supplementary Fig. 4c). Consistent with previous reports^31^, IL-1β treatment of hepatocytes led to a significant reduction in HNF4α protein levels (Fig. 4k). Furthermore, IL-1β-treated primary hepatocytes isolated from marmoset livers presented substantial reductions in A1AT and HNF4α protein levels (Fig. 4l). Manipulation of *Hnf4a* and *Hif1a* gene expression via small-interfering RNA in HepG2 cells revealed that only siHNF4A treatment resulted in decreased mRNA and protein levels of A1AT, an effect not observed with siHIF1A treatment (Fig. 4m and Supplementary Fig. 4d, e). Chromatin immunoprecipitation with sequencing (ChIP-seq) analysis of HepG2 cells revealed significant enrichment of HNF4A^32^ and H3K4me3 (ref.^33^), a histone modification associated with active gene promoters, at the *SERPINA1* promoter region (Fig. 4n). The ChIP–PCR results indicated that the +1,203 bp to +1,331 bp region in the *SERPINA1* promoter, which encompasses the predicted HNF4A binding sites, is crucial for transcriptional activation by HNF4α (Fig. 4o). Collectively, these results indicate that inflammatory stimuli, notably IL-1β, which originates from KCs, promote the downregulation of HNF4α. This leads to the transcriptional repression of A1AT, a mechanism that is conserved across humans, mice and nonhuman primates (Supplementary Fig. 4f).

### MoKCs are key contributors to increased PR3 levels and induce hepatic inflammation in MASH

**Fig. 5:**
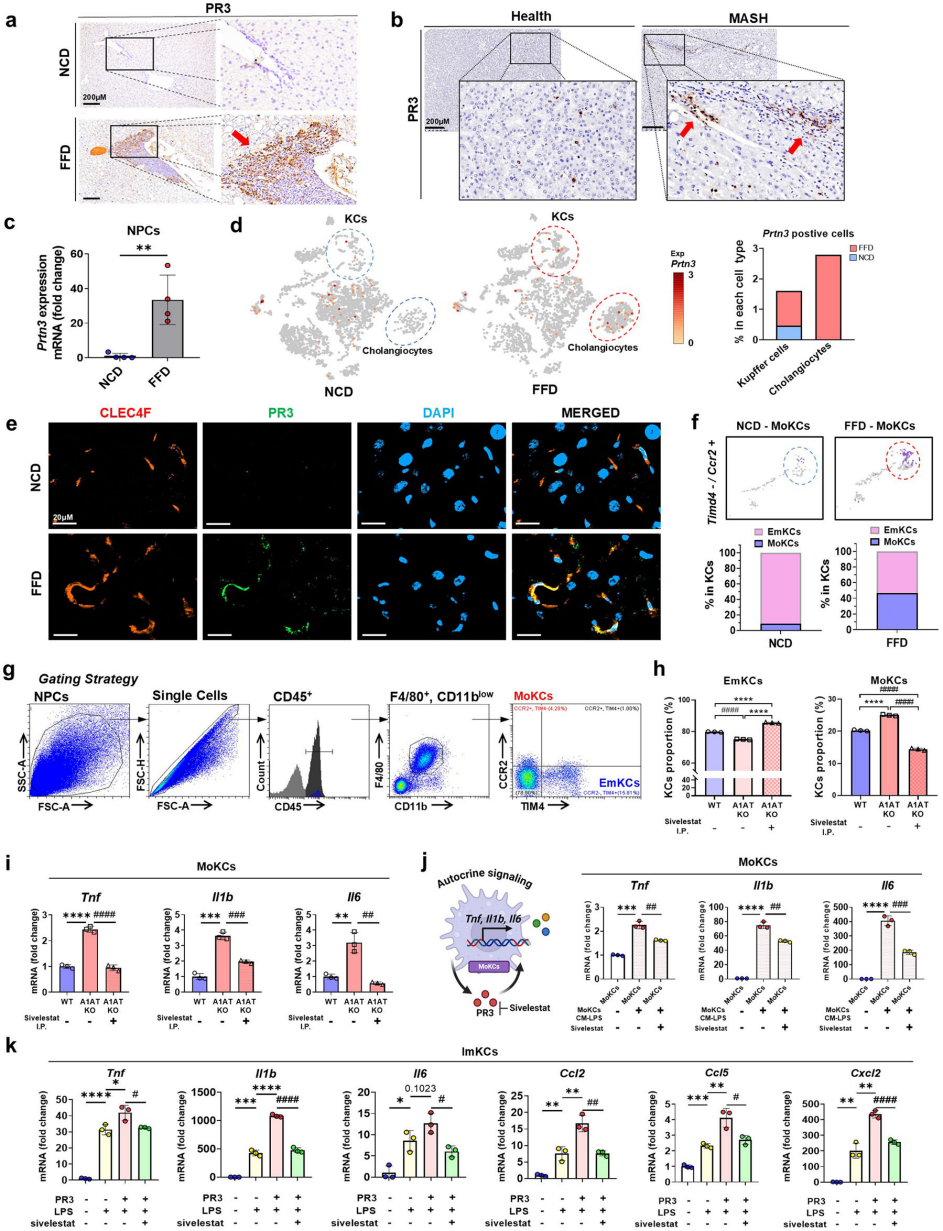
MoKCs play a crucial role in increasing PR3 levels, leading to hepatic inflammation in MASH. **a**,**b**, Representative images of immunohistochemical staining for PR3 expression in liver tissue sections from NCD- and FFD-fed mice (**a**) and healthy controls versus patients with MASH (**b**). **c**, Relative mRNA expression of *Prnt3* in primary NPCs isolated from NCD- and FFD-fed mice. **d**, scRNA-seq analysis of NPCs from NCD- and FFD-fed mice, showing the distribution of *Prtn3* expression across different cell types. **e**, Representative co-immunofluorescence images of liver sections from both NCD- and FFD-fed mice stained for CLEC4F (red, indicating Kcs), PR3 (green) and DAPI (blue, indicating nuclei). **f**, scRNA-seq analysis of EmKCs (Tim4^+^ and Ccr2^−^) and MoKCs (Tim4^−^ and Ccr2^+^) in the Kc pool from NCD- and FFD-fed mice. **g**, Flow cytometry gating strategy for identifying MoKCs (CD45^+^, F4/80^+^, CD11b^low^, TIM4^−^ and CCR2^+^) and EmKCs (CD45^+^, F4/80^+^, CD11b^low^, TIM4^+^ and CCR2^−^) in liver tissue. **h**, Quantification of EmKC and MoKC percentages in WT, A1AT KO and A1AT KO mice treated with sivelestat. **i**, Relative mRNA expression of proinflammatory genes (*Tnf*, *Il1b* and *Il6*) in MoKCs sorted from WT, A1AT KO and A1AT KO mice treated with sivelestat. **j**, Relative mRNA expression of proinflammatory genes (*Tnf*, *Il1b* and *Il6*) in MoKCs stimulated with CM (CM-control, CM-LPS or CM-LPS + sivelestat). **k**, Relative mRNA expression levels of proinflammatory genes in ImKCs treated with PR3, LPS and Sivelestat. The data are presented as the means ± s.d.; *^,#^*P* < 0.05, **^,##^*P* < 0.01, ***^,###^*P* < 0.001 and ****^,####^*P* < 0.0001 versus the control model. ns, not significant.

We then focused on the source of increased PR3 levels, which may contribute to the pathogenesis of MASH. Consistent with the elevated serum PR3 levels previously observed (Fig. 1h, l), increased hepatic infiltration of PR3-positive macrophages was noted in both FFD-fed mice and patients with MASH (Fig. 5a, b and Supplementary Fig. 5a). NPCs derived from FFD-fed mice presented elevated PR3 mRNA expression (Fig. 5c). scRNA-seq analysis revealed upregulated *Prtn3* expression among KCs and cholangiocytes in NPCs from FFD-fed mice compared with those from NCD-fed mice (Fig. 5d). In addition, we observed an increase in CLEC4F- and PR3-positive KCs in FFD-fed mice (Fig. 5e). Notably, an increase in the replenishment of MoKCs was observed in FFD-fed mice (Fig. 5f and Supplementary Fig. 5b). Flow cytometry analysis of KCs from WT mice revealed that MoKCs, identified as CD45^+^F4/80^hi^CD11b^low^TIM4^−^CCR2^+^ cells, presented higher proinflammatory gene and PR3 expression than EmKCs, identified as CD45^+^F4/80^hi^CD11b^low^TIM4^+^CCR2^−^ cells (Supplementary Fig. 5c, d)^34,35^. Flow cytometry analysis of A1AT KO mice revealed that A1AT deletion led to an increased proportion of MoKCs, with elevated expression of proinflammatory genes and PR3, effects that were abrogated by intraperitoneal injection of sivelestat (Fig. 5g, h and Supplementary Fig. 5e, f). Our results further indicate that PR3 secreted by MoKCs exerts autocrine effects, as PR3-induced CM led to further activation of MoKCs, as evidenced by increased expression of proinflammatory markers (Fig. 5i). This effect was attenuated by sivelestat treatment (Fig. 5j). Exogenous treatment with PR3 increased the expression of proinflammatory cytokines and chemokines in KCs and synergistically increased the levels of LPS-induced proinflammatory cytokines (Supplementary Fig. 5g). Sivelestat attenuated PR3-induced inflammation, indicating that the proinflammatory characteristics of PR3 are due to its proteolytic activity (Fig. 5k).

### PR3 binding target IL-32γ exerts anti-inflammatory effects

**Fig. 6:**
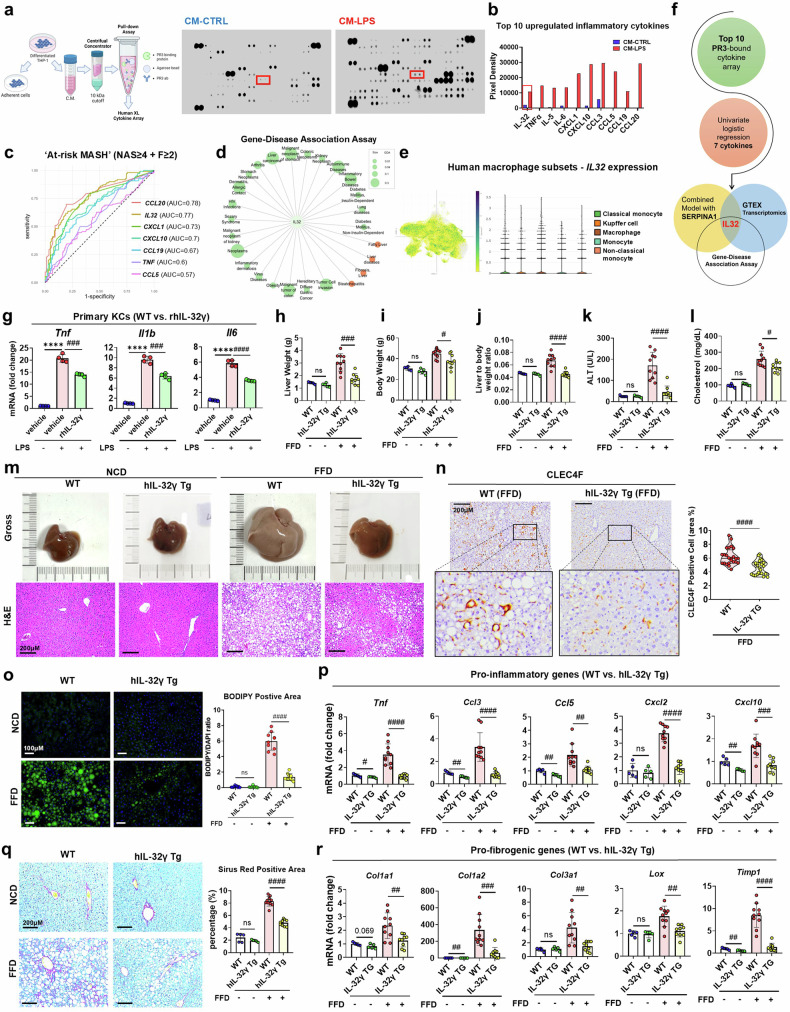
The PR3-targeting protein IL-32γ protects against liver inflammation and fibrosis in mice treated with MASH. **a**, Proteome profiling of a human cytokine array for co-immunoprecipitation of endogenously secreted PR3 in CM from LPS-stimulated (CM-LPS) and nonstimulated (CM-CTRL) THP-1 cells. **b**, A bar graph indicating the average signal intensities of framed spots on the array blots from **a**. Each red bracket in the cytokine array analysis indicates the location of interleukin (IL)-32. **c**, Univariate logistic regression analysis was conducted in a discovery cohort (*n* = 130) to identify patients ‘at-risk’ from MASH based on the gene expression of PR3-bound cytokines. **d**, The gene‒disease network for *IL32* was examined via data from genome-wide association studies that focused on records with a significance level of *P* < 1 × 10^−6^. **e**, Human macrophage subset gene expression of *IL32* based on the Single Cell Portal, Broad Institute. **f**, A Venn diagram illustrating the ability of PR3 to target the inflammatory cytokine IL-32 and its relationship with *SERPINA1* and MASH pathogenesis. **g**, Relative mRNA expression of proinflammatory genes in primary KCs co-stimulated with LPS and recombinant human IL-32γ (rhIL-32γ; 100 ng/ml) for 6 h. **h**–**j**, BWs (**h**), LWs (**i**) and LBW ratios (**j**) of NCD-fed WT mice (*n* = 5), NCD-fed hIL-32γ Tg mice (*n* = 5), FFD-fed WT mice (*n* = 10) and FFD-fed Tg mice (*n* = 9). **k**,**l**, Detection of the serum ALT (**k**) and cholesterol (**l**) levels in each group. **m**, The appearance of freshly collected liver tissues and H&E staining of liver sections from each group. **n**, IHC staining image of CLEC4F-positive KCs in FFD-fed Tg mice and FFD-fed WT mice. **o**, BODIPY-stained liver sections from each group. Representative images of BODIPY-stained sections were selected, and BODIPY-positive areas (green, for lipid accumulation) and DAPI-positive areas (blue, for nuclei and normalization) were measured. **p**, Relative mRNA expression of proinflammatory genes in the hepatic tissues of each group. **q**, Representative images of Sirius red-stained liver sections from each group. Representative images of whole-body liver sections were analyzed for Sirius red-positive areas. **r**, Relative mRNA expression of profibrogenic genes in liver sections. The data are presented as the means ± s.d.; *^,#^*P* < 0.05, **^,##^*P* < 0.01, ***^,###^*P* < 0.001 and ****^,####^*P* < 0.0001 versus the control model. ns, not significant.

Next, we focused on establishing a cause-and-effect relationship between dysregulated PR3 activity and the proteolytic processing of various proinflammatory cytokines^9,10^ (Fig. 5k). Through co-immunoprecipitation using CM from both LPS-stimulated (CM-LPS) and nonstimulated (CM-CTRL) THP-1 cells, followed by human cytokine array analysis, we identified 24 binding partners of PR3 that were more highly expressed in CM-LPS than in CM-CTRL (Fig. 6a and Supplementary Fig. 6a,b). Notably, most of the top ten upregulated proinflammatory cytokines are known to be associated with MASLD progression^21,36–44^ (Fig. 6b). GTex tissue expression analysis revealed that among these cytokines, *IL32* had the highest expression in normal human liver (Supplementary Fig. 6c). To further narrow down the key molecular markers of PR3 binding targets related to steatohepatitis and advanced fibrosis, we conducted univariate logistic regression analysis to predict patients with ‘at-risk MASH’^45^, defined as those with a NAS ≥4 and a fibrosis score of *F* ≥ 2. The Mann‒Whitney *U*-test of the estimated area under the curve (AUC) revealed that seven of the initial ten cytokines in the gene set were significantly expressed (*P* < 0.05) (Fig. 6c). Notably, *CCL20* had the highest AUC of 0.78, but the combined model with *SERPINA1* did not exhibit interactive or synergistic effects (Supplementary Fig. 6d). In contrast, *IL32*, with the second highest AUC of 0.77, had a synergistic effect on *SERPINA1*, which was statistically significant (Supplementary Fig. 6e, f). This synergy was further corroborated in an independent cohort, yielding an AUC of 0.78 (±0.04) (Supplementary Fig. 6f). Additionally, a gene‒disease relationship analysis suggested a link between *IL32* and chronic liver diseases, including MASH (Fig. 6d). In line with these observations, human hepatic *IL32* mRNA expression was positively correlated with the NAS and fibrosis score (Supplementary Fig. 6g). Additionally, transcriptome analysis of liver biopsy samples from patients with MASH, combined with single-cell analysis of human macrophage subsets, revealed a significant positive correlation between *IL32* expression and various immune cell subsets, including KCs (Fig. 6e and Supplementary Fig. 6h, i). Therefore, we explored the functional role of IL-32, which has been identified as a PR3 binding partner and a *Serpina1*-interacting cytokine associated with MASH progression (Fig. 6f). Specifically, we examined IL-32γ, the most active and abundant isoform among the IL-32 variants^46^, and its role in KCs. In IL-32γ-overexpressing immortalized mouse KCs (ImKCs), we found that PR3 binds to endogenously produced IL-32γ (Supplementary Fig. 6j–l). Moreover, treatment of KCs with recombinant IL-32γ protein markedly reduced the expression of proinflammatory cytokines, underscoring the anti-inflammatory role of IL-32γ in KCs (Fig. 6g).

### IL-32γ attenuates hepatic steatosis, inflammation and fibrosis

To investigate the in vivo function of IL-32γ in MASH, human IL-32γ (hIL-32γ) transgenic (Tg) and WT mice were fed either an FFD or an NCD. We first validated the mRNA and protein expression levels of IL-32γ in Tg mice (Supplementary Fig. 6m, n). The BW, LW and LBW ratios of the NCD-fed Tg mice did not significantly differ from those of the NCD-fed WT mice; however, the BW, LW and LBW values of the FFD-fed Tg mice were significantly lower than those of the FFD-fed WT mice (Fig. 6h–j). Moreover, the serum ALT and cholesterol levels were significantly reduced in the FFD-fed Tg mice (Fig. 6k, l). Histological analyses, including H&E staining, IHC and BODIPY staining, revealed that FFD-induced fat accumulation increased the CLEC4F-positive KC population and that steatosis was markedly attenuated in Tg mice (Fig. 6m–o). Consistent with the reduced hepatic steatosis and proliferation of KCs, IL-32γ overexpression improved hepatic inflammatory responses (Fig. 6p). Moreover, liver fibrosis significantly improved in FFD-fed Tg mice, as evidenced by histological staining and quantification of profibrogenic genes (Fig. 6q, r). These results highlight the protective role of IL-32γ as a key target of PR3 in the pathogenesis of MASH.

### Loss of A1AT suppresses the protective effect of IL-32γ on FFD-induced MASH and fibrosis

**Fig. 7:**
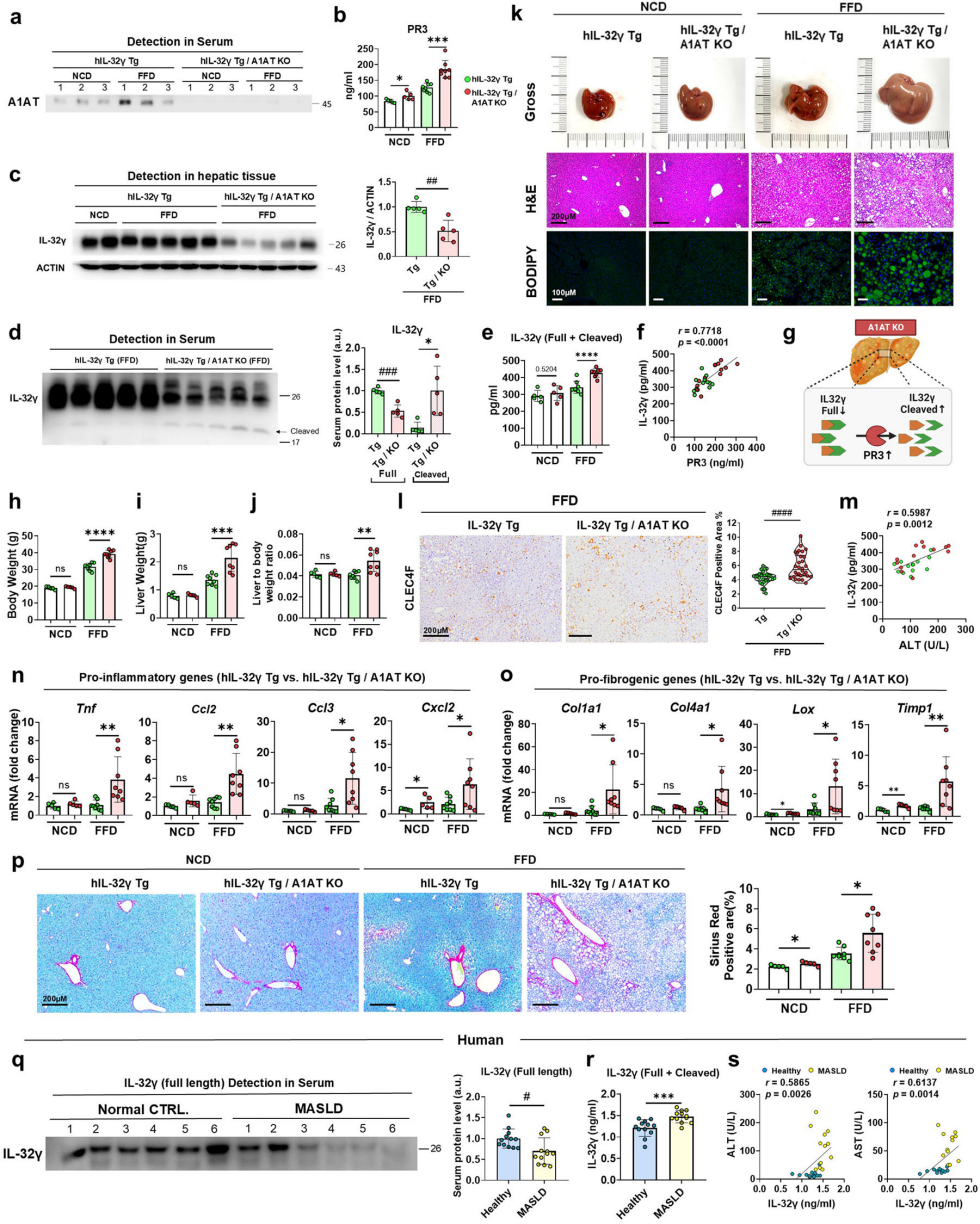
Loss of A1AT blunts the IL-32γ-mediated protective action via proteolytic degradation and cleavage. **a**, Immunoblot analysis of serum A1AT obtained from the NCD- and FFD-fed hIL-32γ Tg mice and the NCD- and FFD-fed Tg/A1AT KO mice. **b**, Serum PR3 concentration of each group measured via ELISA. NCD-fed Tg mice, *n* = 5; NCD-fed Tg/KO mice, *n* = 5; FFD-fed Tg mice, *n* = 8 and FFD-fed Tg/KO mice, *n* = 8 were used. **c**, Hepatic expression of the IL-32γ protein in the NCD- and FFD-fed hIL-32γ Tg mice and the NCD- and FFD-fed hIL-32γ Tg/A1AT KO mice. **d**, Immunoblot analysis of serum IL-32γ in the NCD- and FFD-fed hIL-32γ Tg mice and FFD-fed hIL-32γ Tg/A1AT KO mice. **e**, Serum IL-32γ concentrations in each group were measured via ELISA. **f**, Correlation between the serum level of PR3 and that of IL-32γ in hIL-32γ Tg and hIL-32γ Tg/A1AT KO mice. **g**, Graphical abstract showing PR3-mediated IL-32γ degradation and cleavage in A1AT-depleted conditions. **h**–**j**, BWs (**h**), LWs (**i**) and LBW ratios (**j**) in each group. **k**, Images of freshly collected livers and liver sections from each representative group stained with H&E and BODIPY. **l**, IHC staining image of CLEC4F-positive KCs in FFD-fed Tg mice and FFD-fed Tg/KO mice. **m**, Correlation between the serum levels of IL-32γ and ALT in Tg and Tg/KO mice. **n**,**o**, Relative mRNA expression of proinflammatory genes (**n**) and profibrogenic genes (**o**) in hepatic tissue samples from each group. **p**, Representative images of Sirius red-stained liver sections from each group. Each representative image of a whole-body liver section was analyzed for the Sirius red-positive area. **q**, Detection of cleaved IL-32γ in serum samples from the control group (CRTL.) and in patients with MASLD via western blotting (*n* = 12). **r**, Serum IL-32γ levels in the control group and in patients with MASLD were measured via ELISA. **s**, Correlations between the serum levels of IL-32γ, ALT and AST in the control group and in patients with MASLD. The data are presented as the means ± s.d.; *^,#^*P* < 0.05, **^,##^*P* < 0.01, ***^,###^*P* < 0.001 and ****^,####^*P* < 0.0001 versus the control model. ns, not significant.

To gain mechanistic insights into the A1AT/PR3 imbalance and the functional regulation of IL-32γ in vivo, we crossbred hIL-32γ Tg mice with A1AT KO mice to generate Tg/KO mice. Initially, we verified the in vivo deletion of A1AT and observed an increase in serum PR3 levels (Fig. 7a, b). Notably, dysregulation of the A1AT/PR3 axis led to a reduction in both hepatic expression and serum levels of IL-32γ in FFD-fed Tg/KO mice (Fig. 7c, d). Moreover, increased cleavage of IL-32γ was detected in the serum of FFD-fed Tg/KO mice (Fig. 7d). Using an IL-32γ-specific ELISA, we demonstrated that PR3-mediated proteolytic cleavage of IL-32γ resulted in increased detection of this protein (Supplementary Fig. 7a). The ELISA results further confirmed an increase in the serum IL-32γ level in the FFD-fed Tg/KO mice, along with a positive correlation between the PR3 and IL-32γ serum levels (Fig. 7e, f). These findings indicate an increase in the cleavage of IL-32γ and a corresponding decrease in its full-length form due to excessive PR3 activity in the A1AT-deleted condition (Fig. 7g). FFD-fed Tg/KO mice presented significantly greater BW, LW and LBW ratios (Fig. 7h–j). Histological analysis of these mice revealed markedly aggravated hepatic steatosis, an increased CLEC4F-positive KC population and liver ballooning (Fig. 7k, l and Supplementary Fig. 7b). Similarly, the serum levels of cholesterol, ALT, alkaline phosphatase and amylase were increased in FFD-fed Tg/KO mice (Supplementary Fig. 7c–h). Importantly, we observed a positive correlation between the serum levels of cleaved IL-32γ and ALT (Fig. 7m). FFD-fed Tg/KO mice also displayed markedly severe liver inflammation and fibrosis deposition, as evidenced by increased expression of proinflammatory cytokines and profibrogenic genes and increased Sirius red-positive areas (Fig. 7n–p). To determine the clinical relevance of these findings, we analyzed serum samples from patients with MASLD and a control group with normal liver function. Western blotting and ELISA revealed decreased serum levels of full-length IL-32γ and increased proteolytic cleavage in patients with MASLD (Fig. 7q, r). The increase in cleaved IL-32γ was positively correlated with ALT and AST levels (Fig. 7s). Therefore, these results suggest that A1AT plays a crucial role in protecting against the PR3-mediated proteolysis of IL-32γ. This protective mechanism is essential for maintaining the therapeutic efficacy of IL-32γ in mitigating the progression of MASLD/MASH in both humans and mice.

### The PR3-cleaved C-terminus of IL-32γ is a potent inducer of inflammation and fibrosis

**Fig. 8:**
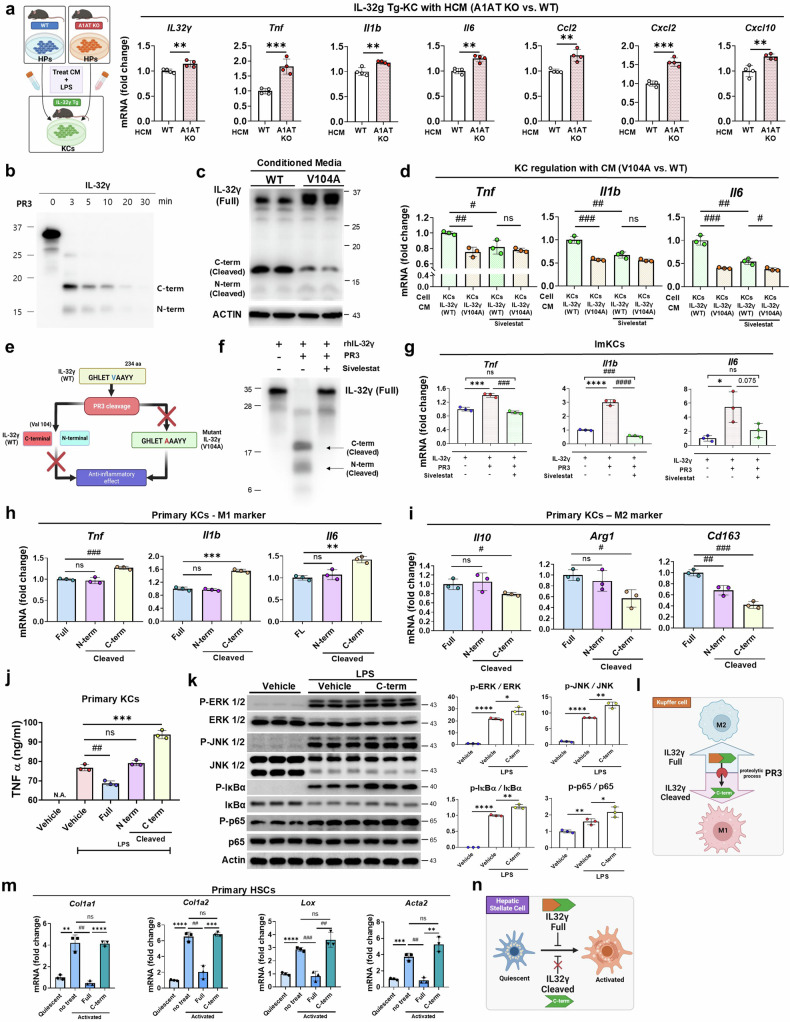
PR3-dependent proteolytic degradation and cleavage of IL-32γ contribute to the activation of KCs and HSCs. **a**, Relative mRNA expression of proinflammatory genes in primary KCs from IL-32γ Tg mice co-stimulated with LPS and A1AT KO HCM or WT HCM. **b**, Immunoblot analysis of PR3-mediated cleavage and degradation of rhIL-32γ in the presence of phosphate-buffered saline (PBS) at different time points at 37 °C. **c**, Proteolytic process of IL-32γ in CM from IL-32γ (WT)- and IL-32γ (V104A)-overexpressing ImKCs stimulated with LPS, as determined via an immunoblot assay. Each CM was concentrated via a concentrator (4,000 rpm for 25 min at 4 °C). **d**, Relative mRNA expression of proinflammatory genes in ImKCs treated with CM from LPS- and sivelestat-treated EV ImKCs, IL-32γ (WT) ImKCs and IL-32γ (V104) ImKCs. **e**, A schematic diagram illustrating the hypothesis that PR3-mediated IL-32γ (WT) cleavage induces immune responses, whereas the anti-inflammatory effect of IL-32γ (V104A) is maintained. **f**, Immunoblot analysis of the cleavage of rhIL-32γ by PR3 in the presence of sivelestat. rhIL-32γ was preincubated with PR3 for 5 min at 37 °C, and the reaction was stopped with a serine protease inhibitor. **g**, Relative mRNA expression of proinflammatory genes in ImKCs treated with rhIL-32γ, PR3 and sivelestat. **h**,**i**, Relative mRNA expression of M1 and M2 markers in primary KCs co-treated with LPS or the full-length (FL)-rhIL-32γ (100 ng/ml), C-terminal (100 ng/ml) or N-terminal (100 ng/ml) domain of IL-32γ. **j**, Detection of TNF in the CM of primary KCs co-treated with LPS, FL-rhIL-32γ (100 ng/ml), the C-terminal domain (100 ng/ml) or the N-terminal domain (100 ng/ml) of IL-32γ. **k**, Immunoblot analysis of p-JNK, JNK, p-ERK, ERK, p-IκB-α, IκB-α, p-p65, p65 and actin protein levels in ImKCs stimulated with LPS with or without the C-terminal cleaved form of IL-32γ. **l**, Graphical figure showing that the cleaved c-terminal domain of IL-32γ by PR3 contributes to the M1 polarization of KCs. **m**, Relative mRNA expression of profibrogenic genes in primary HSCs in the quiescent state (after 1 day of culture) and activated state (after 3 days of culture) stimulated with FL-rhIL-32γ (100 ng/ml) and the C-terminus (100 ng/ml). **n**, The graphical representation illustrates that the cleavage of IL-32γ fails to prevent the activation of HSCs. The data are presented as the means ± s.d.; *^,#^*P* < 0.05, **^,##^*P* < 0.01, ***^,###^*P* < 0.001 and ****^,####^*P* < 0.0001 versus the control model. ns, not significant.

To further investigate the role of IL-32γ in the A1AT/PR3 axis, we co-stimulated primary KCs from IL-32γ Tg mice with LPS and HCM from either A1AT KO or WT mice. As anticipated, primary KCs from Tg mice stimulated with HCM from A1AT KO mice presented increased expression of proinflammatory cytokines (Fig. 8a). Considering the role of PR3 in the proteolytic degradation of IL-32γ (Fig. 8b), we next assessed the impact of blocking the PR3-binding site of IL-32γ through a V104A point mutation. Initially, we verified that ImKCs overexpressing IL-32γ secrete both PR3 and IL-32γ upon LPS stimulation (Supplementary Fig. 8a, b). While overexpressed WT IL-32γ in ImKCs underwent increased degradation following PR3 treatment, V104A-mutated IL-32γ (V104A) demonstrated resistance to PR3-mediated proteolysis (Supplementary Fig. 8c). Moreover, ImKCs overexpressing IL-32γ (WT) produced higher levels of the C-terminal cleaved domain of IL-32γ than those overexpressing IL-32γ (V104A) did (Fig. 8c). We then treated KCs with CM from ImKCs overexpressing either WT IL-32γ (CM-WT) or V104 IL-32γ (CM-V104A). Notably, CM-V104A significantly downregulated the expression of inflammatory genes, and treatment with the PR3 inhibitor sivelestat mitigated the increased inflammatory responses induced by CM-WT (Fig. 8d). These findings suggest that inhibiting PR3 could preserve the anti-inflammatory function of IL-32γ by preventing its proteolytic cleavage (Fig. 8e). We subsequently hypothesized that the increased PR3 levels in MASH may promote proteolytic cleavage of IL-32γ, thus altering its biological function. Indeed, PR3 was found to cleave IL-32γ, leading to elevated expression of proinflammatory cytokines in KCs; however, pretreatment with a PR3 inhibitor effectively reversed these effects, validating our hypothesis (Fig. 8f, g). We then focused on characterizing the functions of the C- and N-terminal domains of IL-32γ cleaved by PR3. After obtaining the C- and N-terminal cleaved domains of IL-32γ at V104 through protein purification, we observed that the C-terminal cleaved form induced M1 polarization of KCs upon LPS stimulation (Fig. 8h–j). This proinflammatory cytokine production by the C-terminal domain was driven by the activation of the NF-κB and MAPK signaling pathways, which are key mediators of the proinflammatory response in KCs (Fig. 8k, l). Furthermore, treatment with the cleaved domains of IL-32γ failed to inhibit the activation of both ONGHEPA1 cells and primary HSCs, whereas full-length IL-32γ significantly reduced profibrogenic gene expression. (Fig. 8m, n and Supplementary Fig. 8d). These findings indicate that the degradation of full-length IL-32γ and the resulting increase in the C-terminal cleaved domain contribute to the proinflammatory environment in KCs and the activation of fibrogenic genes in HSCs.

## Discussion

In our study, we demonstrate the clinical significance of inflammation-induced hepatic downregulation of A1AT and the subsequent upregulation of PR3, leading to the proteolysis of IL-32γ in human MASH. Our findings show that the loss of A1AT increases hepatic PR3 levels, which is driven primarily by the recruitment and expansion of proinflammatory MoKCs. This upregulated PR3 expression, combined with its autocrine effects on MoKCs, amplifies liver inflammation, underscoring the central role of PR3 in MASH progression. Through secretome proteomics, we identified A1AT as the most significantly reduced hepatokine in FFD-induced MASH models, which is consistent with decreased serum A1AT levels in patients with MASH. A1AT deletion further facilitated MoKC replenishment and increased PR3 activity, directly contributing to KC activation and the production of proinflammatory cytokines. Notably, therapeutic interventions with Respreeza, a human serum-derived form of A1AT, and sivelestat, a PR3 inhibitor, significantly reduced the MoKC proportion, liver steatosis, inflammation and fibrosis in mice. These findings suggest that enhancing A1AT expression and inhibiting PR3 activity could be promising therapeutic strategies for managing liver disease progression in patients with MASLD.

In this study, we investigated the hypothesis that the increased activity of PR3 could influence the bioavailability of specific cytokines via proteolytic processing. Notably, IL-32γ is subject to PR3-mediated proteolytic cleavage, which promotes M1 polarization in KCs and fails to prevent the activation of hepatic HSCs. Key inflammatory cytokines, such as IL-1α, IL-1β, IL-18 and IL-33, undergo proteolytic processing by various proteases, thereby modulating their functional roles^47^. IL-32γ, the most biologically active and longest isoform of IL-32, is speculated to contain an immune sensor domain at its C-terminus^46,48^. This domain suggests that IL-32γ might be recognized as a distinct isoform following its cleavage^46^. In our study, we elucidated the in vivo role of human IL-32γ in Tg mice overexpressing hIL-32γ and demonstrated that the overexpression of hIL-32γ confers protection against MASLD by ameliorating lipid accumulation, liver inflammation and steatofibrosis. Our findings collectively underscore the critical role of the A1AT/PR3 imbalance in regulating the severity of hepatic steatosis, inflammation and fibrosis through proteolytic modulation of IL-32γ.

Several studies support the notion that decreased circulating A1AT could cause serious liver damage owing to dysregulated serine proteases in patients with A1AT deficiency, who reportedly experience liver and lung disease due to nonsecretory mutated A1AT and poorly regulated serine proteases^5,6^. Accumulating evidence suggests that excessive and poorly regulated protease activity is strongly associated with inflammatory diseases and is well characterized in patients with MASLD^49–51^. According to DNA microarray analysis and transcriptomics of tissue samples from patients with MASLD and obesity, *IL-32* is a deregulated gene that is correlated with NAS, insulin resistance and aminotransferase levels^20,21^. Moreover, treatment with A1AT prevents the binding of IL-32γ to the PR3 membrane to induce bone marrow inflammation^52^. Accumulating evidence suggests that IL-32γ plays a pivotal role in inflammation and immune responses^19,52,53^ and that the A1AT/PR3 axis regulates the immunomodulatory function of IL-32γ. Consistent with these findings, we demonstrated an important anti-inflammatory effect of A1AT/PR3 axis-mediated IL-32γ on KC and HSC activation and the progression of MASLD.

Previous research has predominantly linked the diminished secretion of hepatokines to the UPR and ER stress^54,55^. However, emerging evidence suggests that an alternative pathway is mediated by inflammatory cytokines^56,57^. In our study, we observed a consistent decrease in the secretion of A1AT in the presence of elevated levels of inflammatory cytokines. This correlation was evident in humans, primates and mouse models. Further analysis revealed that inflammatory cytokines led to the transcriptional downregulation of these hepatokines through the transcription factor HNF4α. Notably, this downregulation was independent of the palmitic acid and H_2_O_2_ associated with the UPR and ER stress in MASLD^29^, suggesting a distinct regulatory mechanism. Through targeted gene expression studies, we demonstrated that the presence of inflammatory cytokines directly impacts the transcriptional machinery governing A1AT production. In contrast to the widely accepted notion that the UPR and ER stress are the primary drivers of decreased hepatokine secretion, our findings highlight the pivotal role of inflammatory cytokines in modulating hepatokine transcription independently. These insights not only challenge the traditional understanding of hepatokine regulation, but also open new avenues for exploring how inflammatory cytokines can selectively alter hepatokine profiles, with potential implications for metabolic diseases. Given these findings, further research is needed to delineate the specific signaling pathways through which inflammatory cytokines exert their transcriptional control over hepatokine production.

Our study utilized Tg mice overexpressing human IL-32γ as a model to investigate the function of human IL-32γ in the context of MASLD. This model has been meticulously developed to bridge the gap in understanding the mechanistic role of IL-32γ in MASH, a critical aspect not yet fully elucidated in human studies. The PiZ Tg mouse strain, which expresses human Glu342Lys-mutated A1AT transgenes, has been used to study the pathogenesis of A1AT deficiency, given the accumulation of A1AT protein aggregates and hepatocyte death^58^. Considering these confounding effects in PiZ Tg mice, investigations of the precise function of native A1AT in liver-specific pathophysiology are limited. Nevertheless, recent work using CRISPR technology has successfully targeted five paralogs of the *Serpina1* gene in mice and led to the development of a *Serpina1a–e* null mouse model^59^. This model was used in the present study to mimic the downregulation of both A1AT mRNA and protein expression observed in patients with MASLD and in FFD-fed mice.

Despite these findings, our study has several limitations. We did not clarify the mechanism underlying the impact of IL-32γ on FFD-induced weight gain and lipid accumulation. Furthermore, we focused on the role of secreted IL-32γ, which can be modified by the exogenous factor PR3. However, intracellular IL-32 has been shown to regulate cell proliferation, differentiation and mitochondrial metabolism in malignant plasma cells^60^. Therefore, future studies to explore the molecular mechanism underlying the endogenous role of IL-32γ in hepatocytes and de novo lipogenesis and to analyze the cell type-specific role of endogenously expressed IL-32γ during the progression of MASLD are needed.

In summary, our study demonstrated that manifestations of hepatic inflammation in MASH, such as increased hepatic IL-1β levels, contribute to the downregulation of circulating A1AT through the transcription factor HNF4α. This downregulation enhances the replenishment of proinflammatory MoKCs, increases PR3 activity and leads to proteolytic modulation of the anti-inflammatory and antifibrogenic cytokine IL-32γ. Thus, targeting the A1AT/PR3/IL-32γ axis could represent a strategy for treating MASH.

## Supplementary information

Supplementary Information
