# Supplementary material for: A1AT dysregulation of metabolically stressed hepatocytes by Kupffer cells drives MASH and fibrosis
Source: Exp Mol Med. 2025 Feb 12;57(2):450–65. doi: 10.1038/s12276-025-01408-1 (PMC11873038; doi:10.1038/s12276-025-01408-1)
Supplement: Supplementary file 1 — Supplementary Information [file 12276_2025_1408_MOESM1_ESM.pdf]

Supplementary Materials for

A1AT dysregulation of metabolically stressed hepatocytes by Kupffer cells drives MASH and fibrosis

Jeong-Su Park[1,†], Jin Lee[2,†] , Feng Wang[1,†], Hwan Ma[1,†], Zixiong Zhou[3], Yong-Sun Lee[4], Kwangyeon Oh[1], Haram Lee[8], Guoyan Sui[1], Sangkyu Lee[5], Yoon Mee Yang[6], Jang-Won Lee[7], Yong-Ha Ji[7], Chun-Woong Park[1], Hwan-Soo Yoo[1], Bang-Yeon Hwang[1], Sang-Bae Han[1], Nan Song[1], Soohwan Oh[8], Bumseok Kim[9], Ekihiro Seki[10], Jin Tae Hong[1,*,#], Yoon Seok Roh[1,*,#]

Correspondence to: ysroh@cbnu.ac.kr and jinthong@chungbuk.ac.kr

This file includes:

Materials and Methods
Supplementary Figures 1 to 8
Raw Data from Secretome Proteomics Analysis
Supplementary Tables 1 and 2

Supplementary Materials and Methods

Sex as a Biological Variable

Animal Study

In our study using C57Bl/6 mice to explore chronic fatty liver disease, sex was identified as a crucial variable, with male mice showing more susceptibility to the disease due to diet-induced weight gain, reduced locomotion with high-fat diets, and impaired glucose tolerance[1,2]. These findings highlight the importance of considering sex in the study of disease mechanisms, though the protective factors in female mice also merit attention.

Human Study

The examination of human serum and tissue samples included both male and female subjects without finding significant sex-based differences in disease markers. This suggests that, within the scope of our analysis, chronic fatty liver disease manifests similarly across sexes, emphasizing the value of inclusive research designs to capture the full spectrum of disease impact.

Primary hepatocytes (HPs) isolation from murine liver

Mice were anesthetized with 30 mg/kg tiletamine/zolazepam (Zoletil®; Vibrac Laboratories, Cat. 8E3HA), and a catheter (24G) was inserted into the inferior vena cava (IVC). The liver was perfused with 30 ml of isolation buffer, comprising 138 mM NaCl (Sigma-Aldrich, Cat. 7647-14-5), 5.4 mM KCl (Sigma-Aldrich, Cat. 7447-40-7), 10 mM HEPES (Sigma-Aldrich, Cat. 7365-45-9), 4.2 mM NAHCO3 (Sigma-Aldrich, Cat. 144-55-8), 0.6 mM NaH2PO4.H2O (Sigma-Aldrich, Cat. 10049-21-5), 0.8 mM Na2HPO4 (Sigma-Aldrich, Cat. 7558-79-4), 0.5 mM EGTA (Sigma-Aldrich, Cat. 99590-86-0), and 5 mM glucose (Sigma-Aldrich, Cat. 50-99-7). The perfusion solution was maintained at 40°C using a Masterflex L/S easy-load II (Cole-Parmer Instrument Co., Cat. EW-77200-60). Subsequently, the liver was perfused with 75 ml of an enzyme buffer solution containing collagenase type 1 (650 µg/ml; Worthington, Cat. LS004197) and collagenase P (50 µg/ml; Roche, Cat. 11213865001). Once successful cannulation was confirmed, a cut was made at the hepatic portal vein to drain the perfusate. The liver was dissected after perfusion was completed, minced well, and filtered through a 100 µm filter; after two washes in enzyme buffer solution, the hepatocytes were resuspended in a complete M199 medium.

Primary hepatocytes (HPs) Isolation from marmoset liver

Fresh marmoset liver was obtained from KBIO HEALTH OSONG Medical Innovation Foundation (KBIO-IACUC-2023-044-6). For the isolation of primary marmoset hepatocytes, the liver, including the portal vein, was carefully excised. The liver was then perfused with 100 ml of an isolation buffer containing 1 mg/ml collagenase type I for 10 minutes to facilitate enzymatic digestion. After perfusion, the liver tissue was dissected, finely minced, and passed through a 100 µm filter to obtain a cell suspension. The hepatocytes were washed twice with the enzyme buffer solution to remove any residual debris. Finally, the isolated hepatocytes were resuspended in complete M199 medium for further use.

Primary hepatic stellate cells (HSCs) and non-parenchymal cells (NPCs) isolation

Mice were anesthetized with 30 mg/kg tiletamine/zolazepam, and a 27G catheter was inserted into the IVC. The liver was perfused with 30 ml of EGTA solution maintained at 40°C using Masterflex L/S easy-load II. Thereafter, the liver was sequentially perfused with 30 ml of an enzyme buffer solution containing pronase (100 µg/ml; Roche, Cat. 11459643001) and 25 ml of an enzyme buffer solution containing collagenase type 1 (250 µg/ml). Once successful cannulation was confirmed, a cut was made at the hepatic portal vein to allow for drainage of the perfusate. The liver was dissected following complete perfusion and incubated for 20 min in secondary digestion solution (enzyme buffer solution containing collagenase type 1 [500 µg/ml], pronase [500 µg/ml], and DNase1 [20 µg/ml; Biosesang, Cat. DF1027-250-00). The digested liver solution was centrifuged, and the supernatant was added to a new 50 ml tube with a cell strainer (70 µm). The mixture was centrifuged and resuspended in 10 ml of washing solution containing DNase I. The volume was made up to 30 ml with washing solution, followed by the addition of 15 ml of 34.5% Nycodenz solution (final concentration, 11.5%; Alere Technologies AS, Cat. 1002424). Then, 14.5% Nycodenz solution was prepared, and 4 ml was transferred into six 15 ml tubes. Subsequently, 7.5 ml of cell-Nycodenz suspension was placed onto the 14.5% Nycodenz solution, and the cell-Nycodenz suspension was gently covered with 1 ml of washing solution. The prepared samples were centrifuged at 3000 rpm for 30 min (4°C) without breakage. Then, the first layer (HSCs) of clear washing buffer and the second layer (NPC) were collected and transferred to new 50 ml tubes, and after two washes in washing buffer, HSCs were resuspended in filtered DMEM medium, as described below. NPCs were resuspended in filtered MACS buffer containing 0.5% bovine serum albumin (BSA) and 2 mM EDTA.

Purification of F4/80+ KCs

For F4/80 positive selection, NPCs were incubated with F4/80 antibodies (Invitrogen, Cat. 13-4801-82) at 4°C for 30 min. Cells were washed with MACS buffer twice, followed by the addition of anti-biotin MicroBeads (Miltenyi Biotec, Cat. 130-090-485). Subsequently, cells were incubated at 4°C for another 15 min, followed by positive selection with the LS column and cell collection in the column. KCs were washed twice with MACS buffer and resuspended in a filtered RPMI medium.

RNA extraction and real-time polymerase chain reaction

RNA was extracted from liver tissues and cells using the Hybrid-R kit (Geneall, Cat. 305-101), according to the manufacturer's instructions. The extracted RNA was subjected to reverse transcription using the High-Capacity cDNA Reverse Transcription kit (Applied Biosystems, Cat. 4368813), followed by PCR using a CFX Connect Real-Time PCR System (BIO-RAD). Target gene expression was normalized to the GAPDH expression level, which was used as the internal control. The primers used for all target genes are listed in Supplementary Table 1.

Western blotting and immunoprecipitation

All liver tissue and cell samples were homogenized in radioimmunoprecipitation assay buffer with protease and phosphatase inhibitors (Atto, Cat. WSE-7420). To detect cleavage of IL-32γ by PR3, we employed recombinant proteins for IL-32γ (YbdY, Cat. REC101) and proteinase 3 (Athens Research & Technology, Cat. 16-14-161820). Western blot samples were centrifuged at 12,000 rpm for 10 min. For human serum sample, we utilized Pierce™ Albumin Serum Depletion Kits (Thermo Fisher Scientific, Cat. 89875) to remove excessive albumin and IgG in the sample according to manufacturer's protocol. The protein concentration was measured using the Pierce BCA Protein Assay Kit (Thermo Fisher Scientific, Cat. 23225). Western blot samples were separated by sodium dodecyl sulfate-polyacrylamide gel electrophoresis (SDS-PAGE), which were subsequently transferred to PVDF membranes. The membranes were blocked with 5% skim milk for 1 h at room temperature or with 5% BSA overnight at 4°C. Primary antibodies used were ACTIN (1:1000; Merck, Cat. MAB1501), GAPDH (1:1000; Merk, Cat. AB2302), IL-32 (1:1000; YbdY, Cat. PAB102), PR3 (1:1000; Santa Cruz, Cat. sc-74534), and A1AT (1:1000; ABclonal,

Cat. A1015), along with secondary antibodies. Membrane images were captured using Amersham imager 600 and quantified using ImageJ. For immunoprecipitation, the control and LPS-stimulated CM of THP-1 cells were incubated with the primary antibody of PR3 (Santa Cruz, Cat. sc-74534) overnight at 4°C. The antibody-conjugated samples were pulled down using protein A/G plus agarose (Santa Cruz, Cat. sc-2003). Each pull-down sample was analyzed using the Proteome Profiler Human XL Cytokine Array kit (R&D Systems, Cat. ARY022B). The antibody used for all target proteins are listed in Supplementary Table 2.

Plasmid, siRNA, and cell lines

The IL-32γ plasmid was point-mutated to alanine at the valine 104 site to generate the IL-32γ (V104A) plasmid. pcDNA3.1+ was used as an internal control vector. Hif1a, Hnf4a, and negative siRNA were purchased from Integrated DNA Technologies. All plasmid and siRNA transfections were performed using Lipofectamine 3000 (Invitrogen, Cat. L3000001). The immortalized mouse KC line was purchased from Sigma-Aldrich (Cat. SCC119). The HepG2 cell line was purchased from the Korean Cell Line Bank, Korea (KCLB No. 88065). ONGHEPA1 [3], mouse hepatic stellate cell line, was provided by Osteoneurogen, Inc.

RNA-seq and data analysis

For murine RNA sequencing results of primary hepatocytes, a cDNA library was constructed using the QuantSeq 3' mRNA-Seq Library Prep Kit (Lexogen) according to the manufacturer's instructions. Briefly, each RNA sample and oligo-dT primer containing an Illumina-compatible sequence at its 5' end was hybridized, followed by reverse transcription. After degradation of the RNA template, second-strand synthesis was initiated by a random primer containing an Illumina-compatible linker sequence at its 5' end. The double-stranded DNA library was purified using magnetic beads. For cluster generation, the library was amplified to add complete adapter sequences. The final product of the library was purified using PCR components. High-throughput sequencing was performed as single-end 75 sequencing using a NextSeq 550 (Illumina).

QuantSeq 3 mRNA-Seq reads were aligned using Bowtie2. Bowtie2 indices were either generated from the genome assembly sequence or representative transcript sequences for alignment to the genome and transcriptome. The alignment file was used to assemble transcripts, estimate their abundance, and detect the differential gene expression. Differentially expressed

genes were determined based on counts from unique and multiple alignments using coverage in Bedtools. The read count data were processed using the TMM+CPM normalization method using EdgeR within R and Bioconductor. Gene classification was based on searches performed using DAVID (http://david.abcc.ncifcrf.gov/) and Medline databases (http://www.ncbi.nlm.nih.gov/). Data mining and graphic visualization were performed using ExDEGA (Ebiogen Inc.).

GEO database analysis

We explored the Gene Expression Omnibus (GEO) datasets related to human MASLD patients on the NCBI database. We analyzed datasets GSE48452, GSE61260, and GSE135251, each of which contained comprehensive patient histories and pivotal pathological indicators of MASH, including fat, inflammation, fibrosis, and NAS. Our analysis focused on patients aged below 70 years. We excluded data from patients who had undergone bariatric surgery, and control liver tissue samples that exhibited inflammation or fibrosis were also omitted from the study.

ChIP seq analysis

The published ChIP-seq data sets for HNF4A (from GSM469863 and GSM469864) and H3K4me4 (GSM2534178 and GSM2534179) were downloaded from GEO database. Each sequencing read was aligned to the hg38 assembly using Bowtie2. The ChIP-seq peaks were identified and visualized by preparing custom tracks on the University of California, Santa Cruz, (UCSC) genome browser using HOMER.

Flowcytometry sorting and analysis

Antibodies were sourced from BioLegend and BD Biosciences. We utilized the following markers and clones: CD11b (M1/70), CD45 (30-F11), F4/80 (BM8), TIMD4 (RMT4-54) and CCR2(SA203G11). Non-parenchymal cells (NPCs) were subjected to density gradient centrifugation using 40% Percoll (1.056 g/mL) at 2000 rpm for 15 minutes, followed by lysis of red blood cells. The collected cell suspensions were stained with the specified antibodies for 20 minutes on ice. After washing, the cells were resuspended in FACS buffer containing 2% FBS, 0.1% sodium azide, and 1 mM EDTA, at a concentration of 1.5 x 10^6 cells/100 µL. Data acquisition was performed using a CytoFLEX SRT cell sorter (Beckman Coulter), and analysis was conducted with FlowJo software.

Univariate logistic regression and Receiver Operating Characteristic (ROC) analyses

To identify potential binding targets of PR3, data from GSE13525, encompassing 10 controls (healthy individuals) and 206 cases of metabolic dysfunction-associated steatohepatitis (MASLD), was retrieved from the Gene Expression Omnibus (GEO). Individuals from this cohort, totaling 216, with a NAFLD activity score (NAS) of 4 or greater and a Fibrosis score of 2 or greater, were categorized as ‘at-risk MASH’. Utilizing the 10 cytokines present in the dataset, we performed univariate logistic regression and Receiver Operating Characteristic (ROC) analyses. Additionally, the Area Under the Curve (AUC) estimates were evaluated for statistical significance using the Mann-Whitney U test. To explore the synergistic effects of the cytokines with *SERPINA1*, combined models were developed through multivariable logistic regression, specifically analyzing *IL32-SERPINA1* and *CCL20-SERPINA1* gene expression data. To validate the robustness of the model, 1,000-fold bootstrap sampling was applied, maintaining a 6:4 ratio for discovery to validation. The statistical significance of differences in the AUC of the combined model and genes was determined through pairwise t-tests. All statistical computations were executed in R software (version 4.3).

Gene-disease analysis

In order to investigate the clinical effects of *IL32*, gene-diseases relationship analysis (GDA) was conducted using DisGeNET plug-in in Cytoscape software.

Supplementary Figure 1

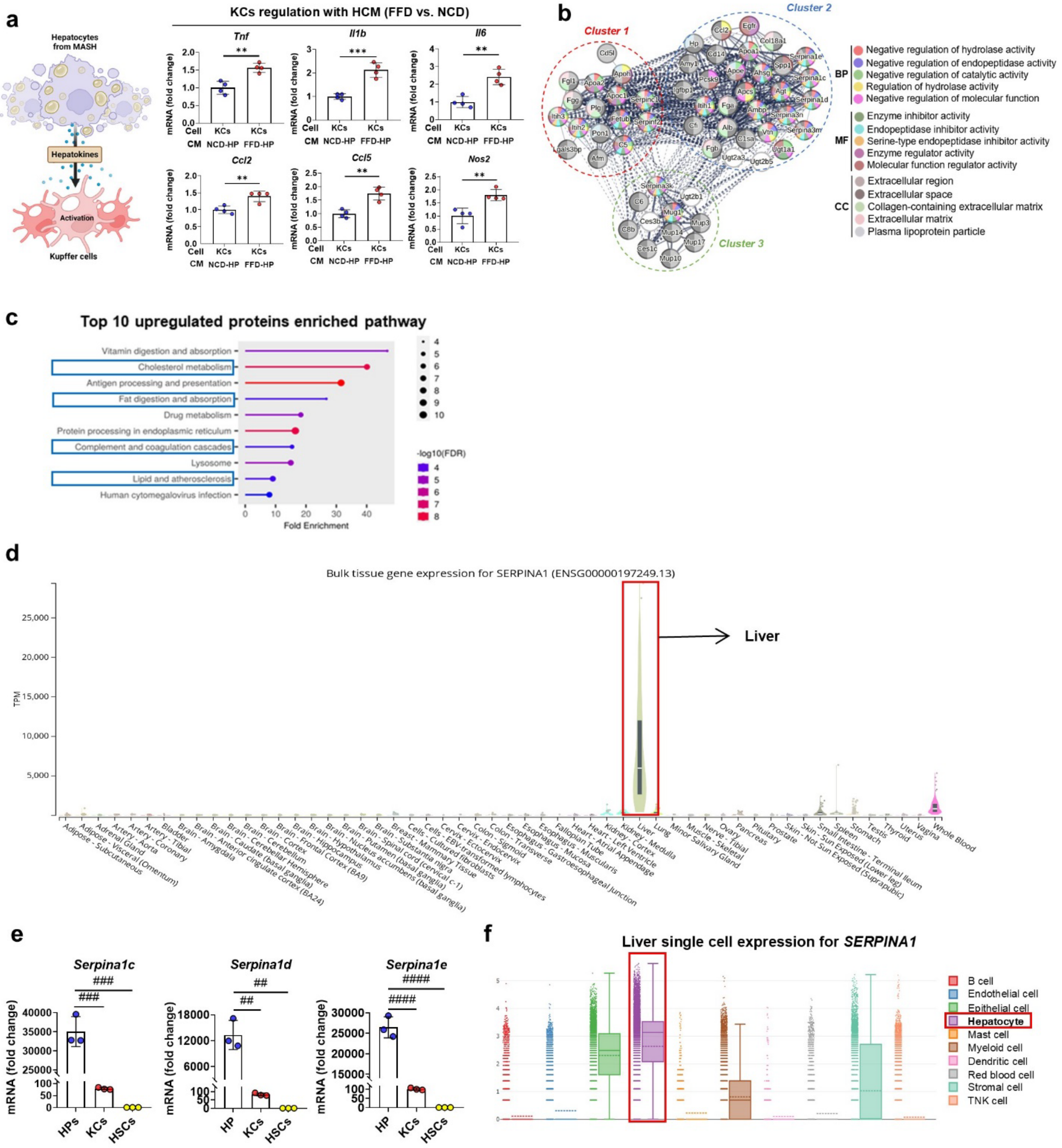

Supplementary Fig. 1 Additional analysis of secretome proteomics and *Serpina1c-e* mRNA expression in murine MASLD models.

a Hepatocyte condition media (HCM) from NCD- or FFD-fed mice are co-treated with LPS on Kupffer cells (KCs), followed by measurement of relative mRNA levels of pro-inflammatory genes. **b** String based protein-protein interaction analysis of differentially secreted proteins. **c**

Enrichment analysis of upregulated proteins revealed in the secretome proteomics study. Enrichment analysis figure was generated by ShinyGO 0.77. **d** Human tissue bulk gene expression of A1AT (*SERPINA1*) in different organs based on GTEx portal. **e** Relative mRNA expression of *Serpinal1c-e* in three major cell types of the liver: hepatocytes (HP), KCs, and hepatic stellate cells (HSCs). **f** Human liver single cell gene expression of A1AT (*SERPINA1*) based on Single Cell Portal, Broad Institute. Data are mean ±SD; *, # P<0.05, **, ## p<0.01, ***, ### p<0.001 and ****, #### p<0.0001 vs. control model. ns, not significant.

Supplementary Figure 2

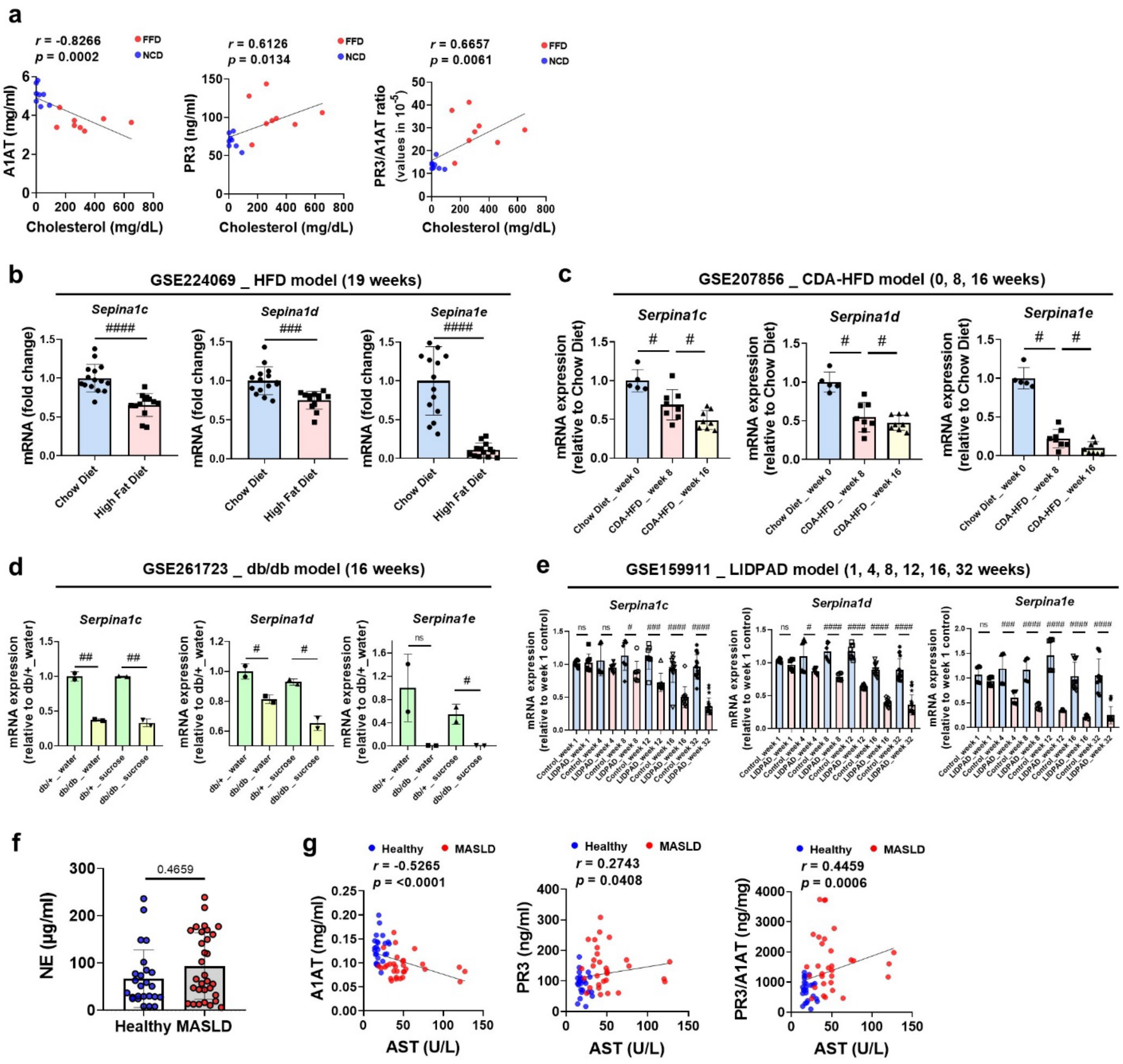

Supplementary Fig. 2 Additional analysis of A1AT (*SERPINA1*) expression in control groups (healthy) and patients with MASLD.

a Correlation of A1AT, PR3, and PR3/A1AT ratio with serum cholesterol levels in NCD- and FFD-fed mice. **b-e** GEO database analysis of different murine MASLD models for *Serpina1c-e* mRNA expression in murine hepatic tissue (GSE224069, GSE207856, GSE261723, GSE159911). **f** Detection of serum levels of neutrophil elastase for the control group (healthy) and patients with MASLD using ELISA. **g** Correlation of A1AT, PR3, and PR3/A1AT ratio with serum AST levels in in the control group (healthy) and patients with MASLD. Data are mean ±SD; *, # P<0.05, **, ## p<0.01, ***, ### p<0.001 and ****, #### p<0.0001 vs. control model. ns, not significant.

Supplementary Figure 3

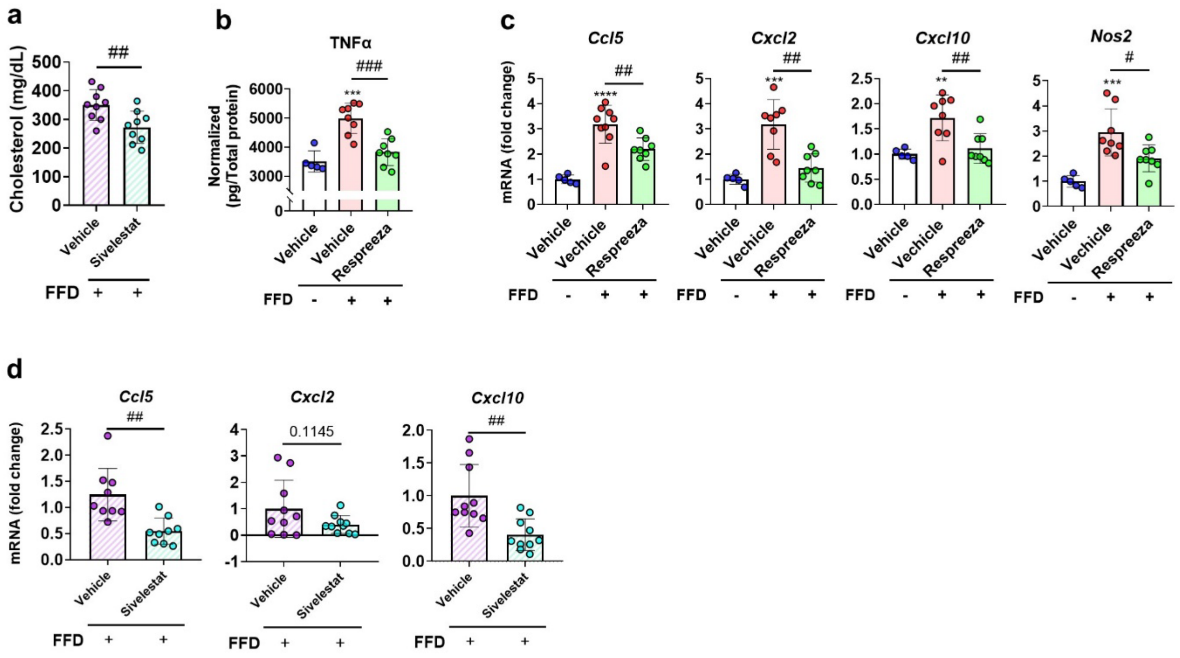

Supplementary Fig. 3 Additional supporting data for *in vivo* studies with Respreeza® and sivelestat.

a Serum cholesterol levels in FFD-fed vehicle mice (n = 10) and FFD-fed sivelestat-treated mice (n = 10). **b** ELISA analysis showing hepatic tissue levels of TNF-α in NCD-fed vehicle (n = 5), FFD-fed vehicle (n = 8), and FFD-fed Respreeza®-treated mice (n = 8). **c** Relative mRNA expression of additional pro-inflammatory genes in hepatic tissues of NCD-fed vehicle, FFD-fed vehicle, and FFD-fed Respreeza®-treated mice. **d** Relative mRNA expression of additional pro-

inflammatory genes in hepatic tissues of FFD-fed vehicle and FFD-fed sivelestat-treated mice. Data are mean ±SD; *, # P<0.05, **, ## p<0.01, ***, ### p<0.001 and ****, #### p<0.0001 vs. control model. ns, not significant.

Supplementary Figure 4

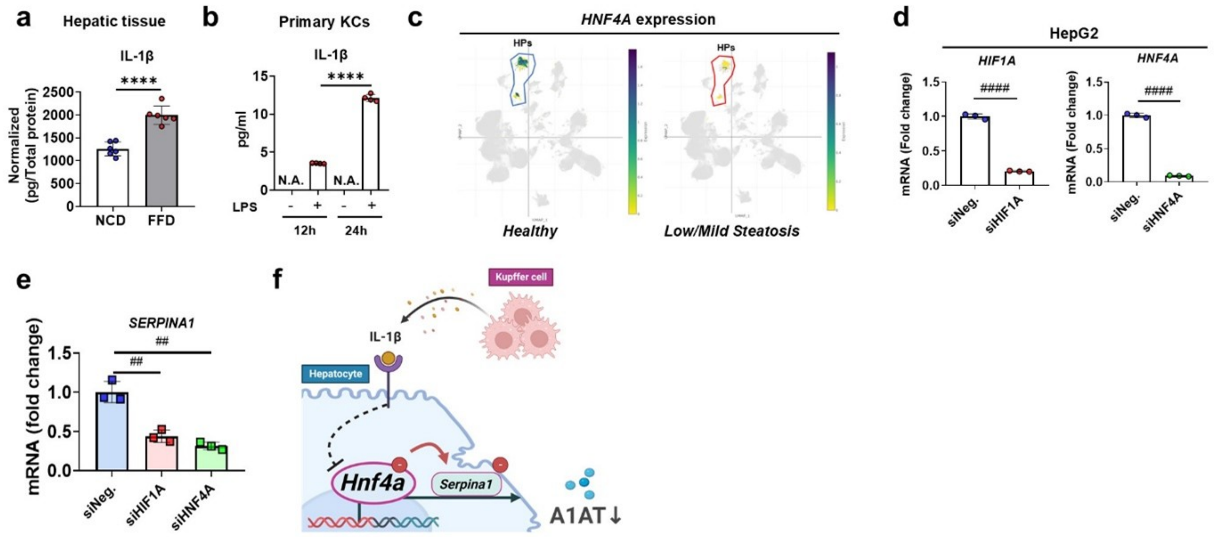

Supplementary Fig. 4 Inflammation mediated by Kupffer cells (KCs) leads to the downregulation of A1AT, which is regulated by the transcription factor HNF4α.

a Hepatic expression of interleukin (IL)-1β protein in tissue from NCD- and FFD-fed mice. **b** Detection of IL-1β in conditioned media of primary KCs stimulated with LPS using Enzyme-linked immunosorbent assay (ELISA). **c** Human liver single cell gene expression of *HNF4A* comparing control group (healthy) and patient diagnosed with low/mild steatosis based on Single Cell Portal, Broad Institute. **d** Relative mRNA expression of *HIF1A*, *HNF4A* in HepG2 cells transfected with siHIF1a, siHNF4a, and siNegative control (siNeg.). **e** Relative mRNA expression of A1AT in HepG2 cells transfected with siHIF1a, siHNF4a, and siNegative control (siNeg.). **f** Graphical depiction of how IL-β downregulates *Hnf4a*, and subsequently, *Serpina1* expression and its production. Data are mean ±SD; *, # P<0.05, **, ## p<0.01, ***, ### p<0.001 and ****, #### p<0.0001 vs. control model. ns, not significant.

Supplementary Figure 5

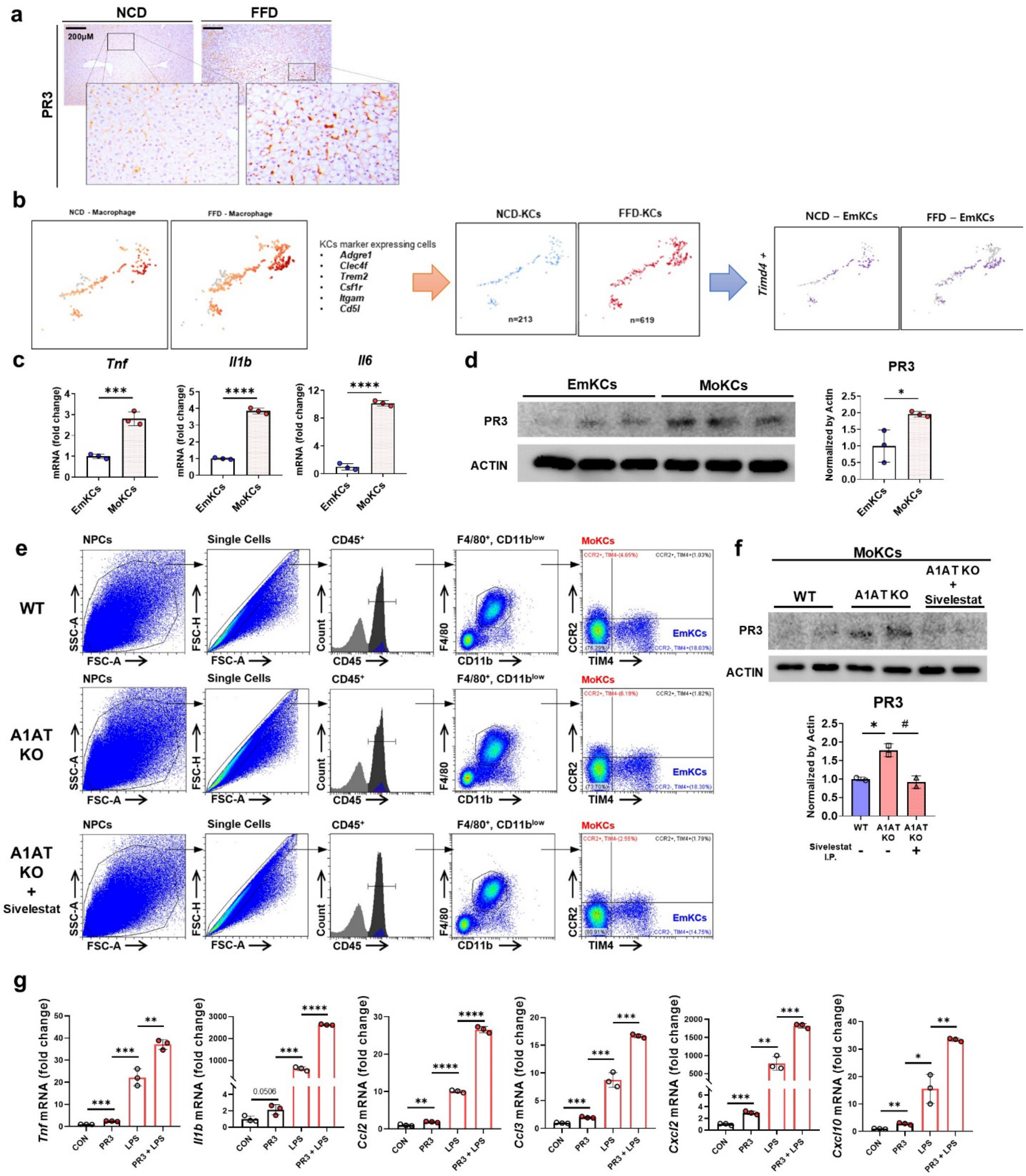

Supplementary Fig. 5 Further characterization of replenishment of PR3 positive cells in FFD-fed mice, and pro-inflammatory role of PR3

a PR3-positive cells in liver tissues of NCD- and FFD-fed mice. **b** scRNA-seq analysis of embryo-derived Kupffer cells (EmKCs) across NPC populations in NCD- and FFD-fed mice. **c** Relative mRNA expression of pro-inflammatory genes in EmKCs and MoKCs. **d** Protein expression of PR3 and Actin in EmKCs and MoKCs. **e** Flow cytometry gating strategy for identifying MoKCs ($CD45^+$, $F4/80^+$, $CD11b^{low}$, $CCR2^+$) , EmKCs ($CD45^+$, $F4/80^+$, $CD11b^{low}$, $TIM4^+$) in WT, A1AT KO, and A1AT KO mice treated with Sivelestat. **f** Protein expression of PR3 and Actin in MoKCs of WT, A1AT KO, and A1AT KO mice treated with sivelestat. **g** Relative mRNA abundance of pro-inflammatory genes in immortalized mouse KCs treated with PR3 and LPS. Data are mean ±SD; *, # P<0.05, **, ## p<0.01, ***, ### p<0.001 and ****, #### p<0.0001 vs. control model. ns, not significant.

Supplementary Figure 6

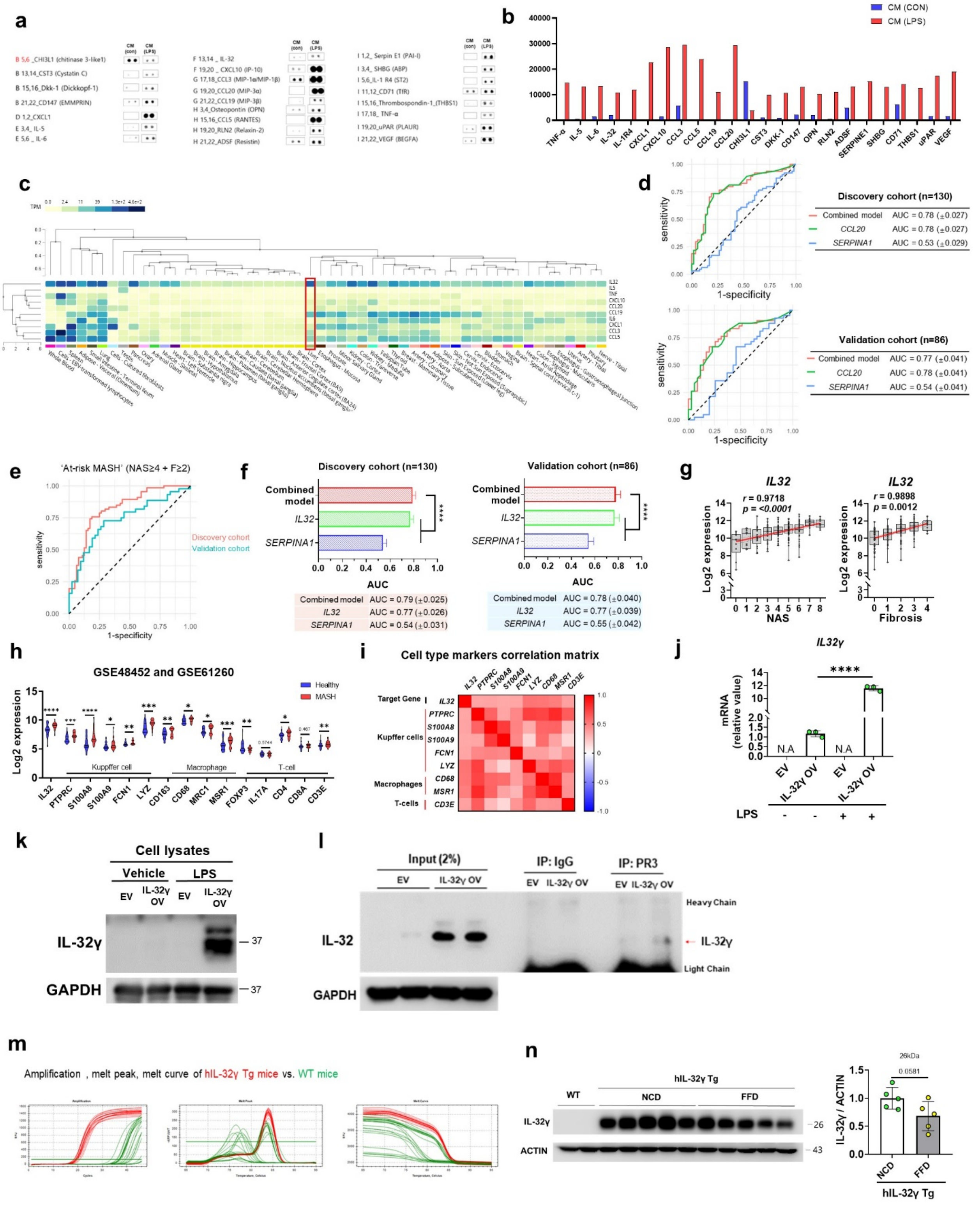

Supplementary Fig. 6 IL-32γ is binding partner of PR3, and further characterization of IL-32γ transgenic mice

a, b Proteome profiling of human cytokine array analysis indicates all cytokines considered binding partners of endogenously secreted PR3 in conditioned media from LPS-stimulated or non-stimulated THP-1 cells (CTRL) and analyzed by framed spots on the array blots. The bar graph indicates the average signal intensities of framed spots on the array blots. **c** Human tissue gene expression of PR3 bound cytokines in different organs based on GTEx portal. **d** The comparison of the combined predictive model with the single gene signatures (*CCL20* and *IL32*) in two independent cohorts (discovery n=130, validation n=86). **e, f** The comparison of the combined predictive model with the single gene signatures (*SERPINA1* and *IL32*) in two independent cohorts (discovery n=130, validation n=86). Bar charts depict the AUC for each score alongside its standard error, as determined through ROC analysis. **g** Human hepatic tissue expression of IL32 in accordance with NAS and fibrosis score using transcriptomic dataset GSE135251. **h, i** Unique cell marker expressions (h) of KC, macrophage and T-cell and correlation matrix (i) between *IL32* (target gene) and other unique cell markers in KCs, macrophages, and T-cells analyzed from GEO databases [GSE48452 and GSE61260]. **j** Relative mRNA expression of *IL32γ* in immortalized mouse Kupffer cells (ImKCs) transfected with IL-32γ and empty vector (EV). **k** Protein expression of IL-32γ in immortalized mouse Kupffer cells (ImKCs) transfected with IL-32γ and EV. pcDNA3.1+ was used as the internal control vector. **l** Co-IP was performed by pull-down of endogenous PR3 in IL-32γ OV ImKCs (IL-32γ OV) and empty vector-transfected ImKCs (EV). **m** Amplification, melt peak, and melt curve of real-time PCR data of hepatic tissue samples from hIL-32γ Tg and wild-type (WT) mice. **n** Hepatic protein expression of IL-32γ in WT, NCD-, and FFD-fed hIL-32γ Tg mice. Data are mean ±SD; *, # P<0.05, **, ## p<0.01, ***, ### p<0.001 and ****, #### p<0.0001 vs. control model. ns, not significant.

Supplementary Figure 7

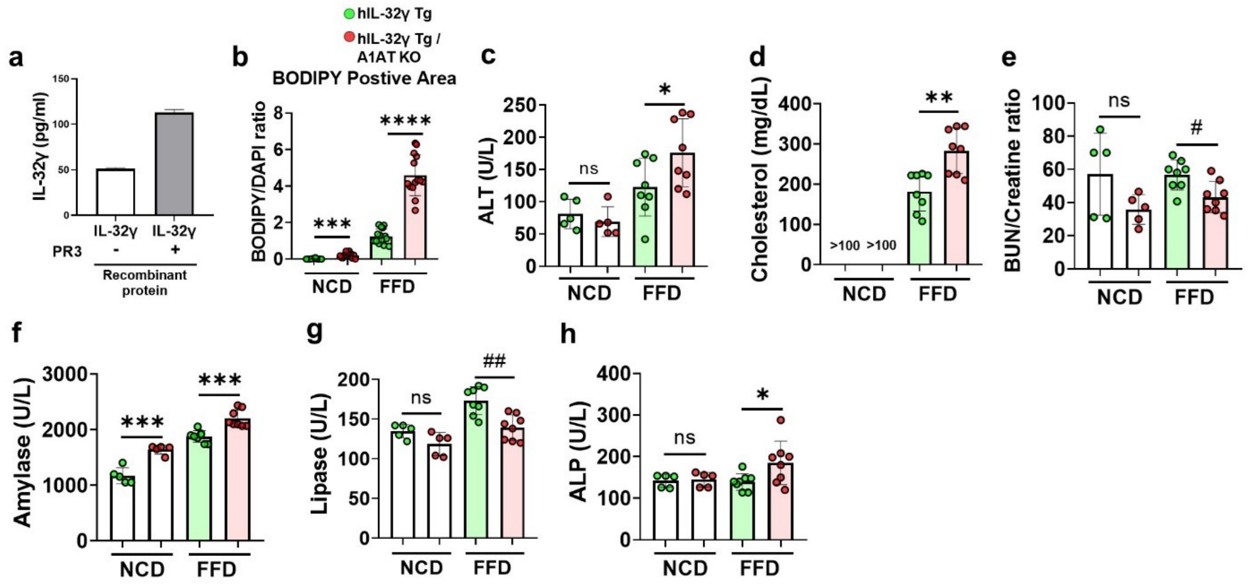

Supplementary Fig. 7 Additional characterization of IL-32γ Tg/A1AT KO mice in FFD model

a ELISA analysis of IL-32γ and PR3-cleaved IL-32γ. **b** Quantification of BODIPY-stained liver sections. Each representative image of a BODIPY-stained section was selected and measured to determine the BODIPY-positive area (green: for lipid accumulation) and DAPI-positive area (blue: for nuclei and normalization). **c-h** Serum levels of ALT (c), Cholesterol (d), BUN/creatine ratio (e), amylase (f), lipase (g), and ALP (h) in NCD-fed hIL-32γ Tg mice (n = 5), NCD-fed hIL-32γ Tg/A1AT KO mice (n = 5), FFD-fed hIL-32γ Tg mice (n = 8), and FFD-fed hIL-32γ Tg/A1AT KO mice (n = 8). Data are mean ±SD; *, # P<0.05, **, ## p<0.01, ***, ### p<0.001 and ****, #### p<0.0001 vs. control model. ns, not significant.

Supplementary Figure 8

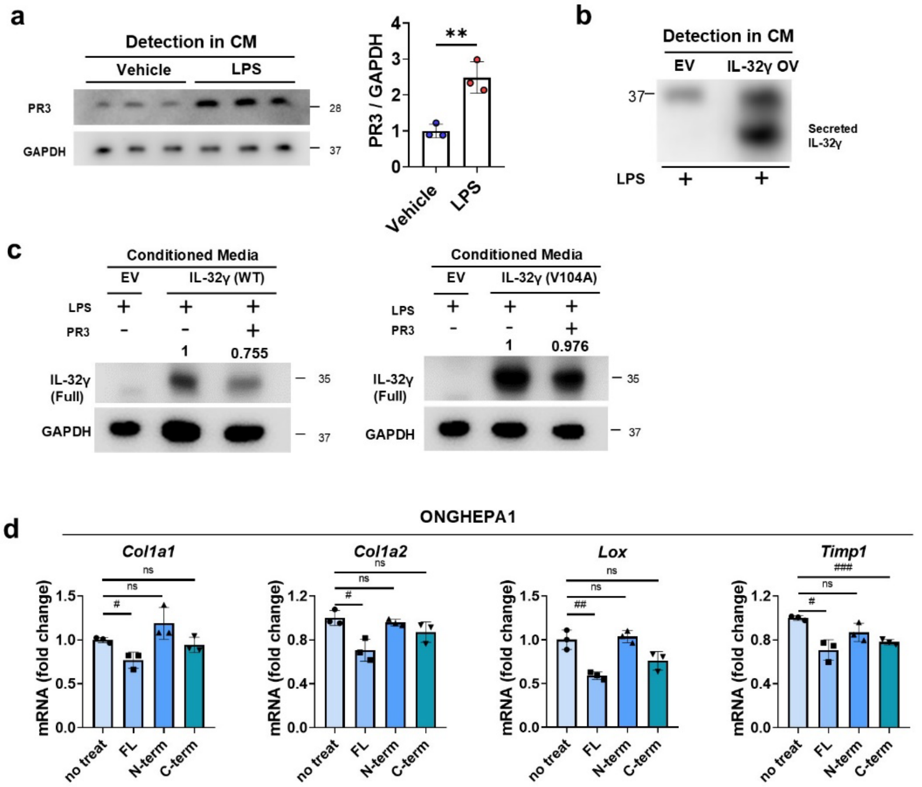

Supplementary Fig. 8 Characteristics of PR3 mediated cleaved domains of IL-32γ. **a** Immunoblot analysis of PR3 secreted in LPS-stimulated ImKCs and non-stimulated ImKCs (vehicle). **b** Immunoblot analysis of secreted IL-32γ detection in cell soup (CM) of IL-32γ overexpressed (OV) ImKCs and EV ImKCs. **c** PR3 degradation analysis of secreted IL-32γ in ImKCs transfected with IL-32γ vector, as determined using an immunoblot assay. PR3 cleavage analysis of secreted IL-32γ in ImKCs transfected with point-mutated vector (valine 104 to alanine), as determined using an immunoblot assay. **d** Relative mRNA abundance of pro-fibrogenic genes in ONGHEPA1 cell lines treated with full-length (FL)-rhIL-32γ (100 ng/ml), C-terminal (100 ng/ml), and N-terminal domain (100 ng/ml) of IL-32γ for 48hours. Data are mean ±SD; *, # $P<0.05$, **, ## $p<0.01$, ***, ### $p<0.001$ and ****, #### $p<0.0001$ vs. control model. ns, not significant.

Raw Data from Secretome Proteomics Analysis

Accesion	Protein name	Gene name	Trial 1 ratio (FFD/NCD)	Trial 2 ratio (FFD/NCD)	Trial 1 p-value	Trial 2 p-value	secreted (1) or not (0)	MEAN
Q9QWK4	CD5 antigen-like	Cd5l	2.04452	4.81592	0.009679	0.009252	1	3.43022
P34928	Apolipoprotein C-I	Apoc1	2.55058	2.67819	0.009507	0.002105	1	2.614385
P06728	Apolipoprotein A-IV	Apoa4	1.05559	2.6784	0.001031	0.001486	1	1.866995
Q547B5	Osteopontin	Spp1	1.95407	1.45526	0.00138	0.007909	1	1.704665
Q8HWB2	Histocompatibility 2, Q region locus 4	H2-Q4	1.3462	1.95739	0.048429	0.012419	1	1.651795
Q07797	Galectin-3-binding protein	Lgals3bp	1.32737	1.95679	9.82E-05	0.002226	1	1.64208
F6ZIS7	Retinoic acid receptor responder	Rarres1	1.4278	1.76577	0.000272	0.002988	1	1.596785
Q3TXU4	Apolipoprotein E	Apoe	1.5042	1.62891	0.004028	0.001568	1	1.566555
P09103	Protein disulfide-isomerase	P4hb	2.35113	0.773452	0.001117	0.002525	1	1.562291
O08601	Microsomal triglyceride transfer protein large subunit	Mttp	2.31102	0.719639	0.00795	0.003473	1	1.51533
P06745	Glucose-6-phosphate isomerase	Gpi	1.62202	1.28758	0.004777	0.007272	1	1.4548
F8WI14	Extracellular matrix protein 1	Ecm1	1.11182	1.77189	0.009406	0.002763	1	1.441855
P09813	Apolipoprotein A-II	Apoa2	1.24134	1.60931	0.012074	0.001904	1	1.425325
E9PV38	Carboxylic ester hydrolase	Ces2g	1.66125	1.18256	3.09E-05	0.001844	1	1.421905
Q00623	Apolipoprotein A-I	Apoa1	1.2522	1.58281	0.005383	0.001355	1	1.417505
Q4FJP7	Monocyte differentiation antigen CD14	Cd14	1.40812	1.37753	0.000902	0.005578	1	1.392825
Q4JFI8	Serum amyloid P-component	Apcs	1.52847	1.24541	0.000126	0.002679	1	1.38694

P08003	Protein disulfide-isomerase A4	Pdia4	2.06898	0.70292	0.007061	0.00327	1	1.38595
Q8BHN3	Neutral alpha-glucosidase AB	Ganab	1.82636	0.937673	0.004281	0.005001	1	1.382017
O88569	Heterogeneous nuclear ribonucleoproteins A2/B1	Hnrnpa2b1	2.05259	0.669707	0.006311	0.034868	1	1.361149
Q5SVU3	C-C motif chemokine 2	Ccl2	1.33839	1.37948	0.006715	0.005532	1	1.358935
Q3U9T8	C-C motif chemokine 9	Ccl9	0.817947	1.86302	0.004169	0.029165	1	1.340484
P14211	Calreticulin	Calr	1.98186	0.653448	0.003006	0.002733	1	1.317654
Q3U6J9	Palmitoyl-protein thioesterase 1	Ppt1	1.77139	0.840694	0.001567	0.003148	1	1.306042
Q9QXT0	Protein canopy homolog 2	Cnpy2	1.95311	0.655238	0.009231	0.002379	1	1.304174
Q922R8	Protein disulfide-isomerase A6	Pdia6	1.6982	0.821612	0.01355	0.016379	1	1.259906
P20029	78 kDa glucose-regulated protein	Hspa5	1.78513	0.731255	0.000795	0.003056	1	1.258193
Q6P5E4	UDP-glucose:glycoprotein glucosyltransferase 1	Uggt1	1.56837	0.898188	0.016507	0.013253	1	1.233279
P01887	Beta-2-microglobulin	B2m	1.04824	1.40447	0.001345	0.000959	1	1.226355
H3BL34	Carboxylesterase 1E	Ces1e	1.79587	0.648494	0.01068	0.007684	1	1.222182
Q03311	Cholinesterase	Bche	1.30015	1.13747	0.009544	0.001829	1	1.21881
P28843	Dipeptidyl peptidase 4	Dpp4	1.53256	0.898168	0.020641	0.002465	1	1.215364
P12265	Beta-glucuronidase	Gusb	1.54351	0.886525	0.007223	0.013645	1	1.215018
Q792Z7	H-2 class I histocompatibility antigen, D-B alpha chain	H2-D1	1.21864	1.19805	0.033602	0.011683	1	1.208345
Q8BK48	Pyrethroid hydrolase Ces2e	Ces2e	1.38495	1.02009	0.001225	0.000505	1	1.20252
O89017	Legumain	Lgmn	1.13079	1.23994	0.01531	0.04793	1	1.185365

Q8QZR4	Out at first protein homolog	Oaf	1.02514	1.33947	0.007585	0.001383	1	1.182305
Q3UAD6	Endoplasmin	Hsp90b1	1.69043	0.666805	0.004507	0.011249	1	1.178618
Q91XL1	Leucine-rich HEV glycoprotein	Lrg1	1.24584	1.10185	0.015154	0.001709	1	1.173845
Q8R164	Valacyclovir hydrolase	Bphl	1.74749	0.577408	0.000349	0.00344	1	1.162449
O88968	Transcobalamin-2	Tcn2	0.795393	1.50625	0.030153	0.005162	1	1.150822
Q8C7G5	Apolipoprotein A-V	Apoa5	0.838456	1.41463	0.009611	0.007446	1	1.126543
A2APX3	Cystatin-C	Cst3	0.954281	1.298	0.006628	0.01072	1	1.126141
Q9JKR6	Hypoxia up-regulated protein 1	Hyou1	1.44871	0.798333	0.020953	0.001179	1	1.123522
Q5I0U6	Serum amyloid A protein	Saa1	1.59493	0.643721	0.026929	0.000345	1	1.119326
Q3TH01	H-2 class I histocompatibility antigen, K-B alpha chain	H2-K1	0.937621	1.29072	0.002633	0.010686	1	1.114171
Q9WUZ9	Ectonucleoside triphosphate diphosphohydrolase 5	Entpd5	1.30454	0.919649	0.022347	0.027467	1	1.112095
P11672	Neutrophil gelatinase-associated lipocalin	Lcn2	1.20755	1.01593	0.002445	0.004427	1	1.11174
H7BX99	Prothrombin	F2	1.08204	1.13842	0.000168	0.003245	1	1.11023
Q8K1I3	Secreted phosphoprotein 24	Spp2	0.853069	1.35567	0.002823	0.003545	1	1.10437
E9Q414	Apolipoprotein B-100	Apob	0.919879	1.28328	0.002713	0.007764	1	1.10158
Q02819	Nucleobindin-1	Nucb1	1.0149	1.18382	0.001996	0.011213	1	1.09936
P33587	Vitamin K-dependent protein C	Proc	0.90856	1.28833	0.004325	0.002915	1	1.098445
Q9ESB3	Histidine-rich glycoprotein	Hrg	0.953366	1.24348	0.005066	0.002115	1	1.098423
Q91V80	Apolipoprotein F	Apof	0.886574	1.3082	0.004196	0.000709	1	1.097387

O70362	Phosphatidylinositol-glycan-specific phospholipase D	Gpld1	1.0999	1.07285	0.007625	0.003022	1	1.086375
Q8VCT4	Carboxylesterase 1D	Ces1d	1.58719	0.583545	0.01015	0.009275	1	1.085368
Q91WU0	Carboxylesterase 1F	Ces1f	1.60072	0.568977	0.004505	0.001427	1	1.084849
Q64726	Zinc-alpha-2-glycoprotein	Azgp1	0.921516	1.23179	0.006852	0.005412	1	1.076653
Q61147	Ceruloplasmin	Cp	0.898239	1.25051	0.001198	0.002404	1	1.074375
A0A0R4J1N3	Apolipoprotein C-III	Apoc3	0.9094	1.22426	0.000414	0.001777	1	1.06683
Q3UCD9	Cathepsin D	Ctsd	0.987811	1.14443	0.001755	0.004405	1	1.066121
Q9WUU7	Cathepsin Z	Ctsz	1.00421	1.12714	0.002953	0.003808	1	1.065675
E9PX70	Collagen alpha-1(XII) chain	Col12a1	1.35219	0.768237	0.000223	0.001867	1	1.060214
Q9CPT4	UPF0556 protein C19orf10 homolog	D17Wsu104e	0.960012	1.15886	0.017761	0.010241	1	1.059436
P10639	Thioredoxin	Txn	1.32175	0.783232	0.006192	0.012414	1	1.052491
Q8BND5	Sulfhydryl oxidase 1	Qsox1	0.907953	1.19383	0.005993	0.001038	1	1.050892
D6RGQ0	Complement factor H	Cfh	0.839653	1.25696	0.001879	0.008347	1	1.048307
P27773	Protein disulfide-isomerase A3	Pdia3	1.3285	0.75978	0.006749	0.012655	1	1.04414
P10605	Cathepsin B	Ctsb	0.869875	1.19917	0.004326	0.000981	1	1.034523
Q9WVJ3	Carboxypeptidase Q	Cpq	1.05932	0.995558	0.001028	0.000874	1	1.027439
O88783	Coagulation factor V	F5	0.875034	1.17705	0.006654	0.008088	1	1.026042
B8JJN0	Complement factor B	Gm20547	0.887767	1.13325	0.000826	0.0007	1	1.010509
Q91WP0	Mannan-binding lectin serine protease 2	Masp2	0.927991	1.09124	0.032667	0.016706	1	1.009616
Q5M9K1	Transthyretin	Ttr	0.907067	1.10892	0.005966	0.002783	1	1.007994
P14847	C-reactive protein	Crp	0.94226	1.07262	0.009911	0.001543	1	1.00744
Q5FW60	Major urinary protein 20	Mup20	0.769705	1.24514	0.005733	0.031282	1	1.007423

P49935	Pro-cathepsin H	Ctsh	1.04392	0.967757	0.006507	0.003102	1	1.005839
Q8VCS0	N-acetylmuramoyl-L-alanine amidase	Pglyrp2	0.821496	1.1887	5.91E-05	0.001672	1	1.005098
Q91YW3	DnaJ homolog subfamily C member 3	Dnajc3	0.963239	1.029	0.00237	0.00041	1	0.99612
Q3UEK1	Mannose-binding protein C	Mbl2	0.785813	1.1987	0.001736	0.003316	1	0.992257
O09159	Lysosomal alpha-mannosidase	Man2b1	0.925814	1.0575	0.000355	0.012346	1	0.991657
Q6S9I0	Kininogen 2	Kng2	0.743674	1.23378	0.000857	0.000741	1	0.988727
Q9R182	Angiopoietin-related protein 3	Angptl3	0.883213	1.09192	0.008657	0.003504	1	0.987567
Q05117	Tartrate-resistant acid phosphatase type 5	Acp5	0.768972	1.20109	0.027139	0.0161	1	0.985031
P01898	H-2 class I histocompatibility antigen, Q10 alpha chain	H2-Q10	0.76126	1.20873	0.005809	0.013927	1	0.984995
Q3TKX1	V-type proton ATPase subunit S1	Atp6ap1	1.02227	0.936351	0.027374	0.013622	1	0.979311
Q91X72	Hemopexin	Hpx	0.867959	1.08764	0.002989	0.001475	1	0.9778
P11087	Collagen alpha-1(I) chain	Col1a1	0.911669	1.03564	0.009289	0.020886	1	0.973655
Q549A5	Clusterin	Clu	0.797368	1.14035	0.004897	0.010489	1	0.968859
A6X935	Inter alpha-trypsin inhibitor, heavy chain 4	Itih4	0.879536	1.05113	0.000739	0.000254	1	0.965333
Q8CIF4	Biotinidase	Btd	0.926877	1.0035	0.027785	0.030957	1	0.965189
Q63880	Carboxylesterase 3A	Ces3a	1.337	0.592476	0.004252	0.005095	1	0.964738
P10493	Nidogen-1	Nid1	0.731994	1.19563	0.008627	0.018837	1	0.963812
E9QK54	Serpin A11	Serpina11	0.998547	0.926358	0.005834	0.002539	1	0.962453
O70570	Polymeric immunoglobulin receptor	Pigr	0.843833	1.06681	0.014318	0.017702	1	0.955322

P01029	Complement C4-B	C4b	0.853586	1.02455	0.008881	0.002736	1	0.939068
Q8R121	Protein Z-dependent protease inhibitor	Serpina10	0.825615	1.04083	0.000748	0.001434	1	0.933223
Q60590	Alpha-1-acid glycoprotein 1	Orm1	0.916524	0.949337	0.005718	0.000677	1	0.932931
Q921I1	Serotransferrin	Tf	0.788954	1.06271	0.00301	0.010643	1	0.925832
Q543M3	Cathepsin L1	Ctsl	0.783794	1.06105	0.001773	0.007485	1	0.922422
P22599	Alpha-1-antitrypsin 1-2	Serpina1b	0.859358	0.984908	0.000348	0.000198	1	0.922133
Q3TBR2	Coagulation factor X	F10	0.818148	1.0229	0.000638	0.002316	1	0.920524
A2AS37	Expressed sequence AI182371	AI182371	0.767041	1.05419	0.006697	0.000563	1	0.910616
P97298	Pigment epithelium-derived factor	Serpinf1	0.8455	0.961631	0.000791	0.001498	1	0.903566
D3YTY9	Kininogen-1	Kng1	0.659818	1.12343	0.001017	0.003157	1	0.891624
P49182	Heparin cofactor 2	Serpind1	0.721604	1.05478	0.002116	0.000904	1	0.888192
Q3TUF3	Calumenin	Calu	0.904857	0.843621	0.00376	0.028054	1	0.874239
P07361	Alpha-1-acid glycoprotein 2	Orm2	0.731972	1.00245	0.000385	0.00518	1	0.867211
G5E899	Plasminogen activator inhibitor 1	Serpine1	0.778116	0.94862	0.007563	0.001127	1	0.863368
Q8K2I4	Beta-mannosidase	Manba	0.884743	0.840665	0.000199	0.002576	1	0.862704
B7ZNJ1	Fibronectin	Fn1	0.679054	1.03397	0.004929	0.004785	1	0.856512
P06683	Complement component C9	C9	0.780983	0.92925	0.008118	0.040377	1	0.855117
P05367	Serum amyloid A-2 protein	Saa2	1.16722	0.532452	0.001156	0.006923	1	0.849836
P01027	Complement C3	C3	0.743348	0.947018	0.001543	0.001518	1	0.845183
E9QQ18	Proteoglycan 4	Prg4	0.679972	1.00566	0.012422	0.000655	1	0.842816
Q9JHH6	Carboxypeptidase B2	Cpb2	0.855405	0.827508	0.014615	0.004602	1	0.841457
Q80YC5	Coagulation factor XII	F12	0.700093	0.979223	0.016091	0.001723	1	0.839658

P97290	Plasma protease C1 inhibitor	Serping1	0.733058	0.942248	0.006652	0.014684	1	0.837653
Q9D1Q6	Endoplasmic reticulum resident protein 44	Erp44	1.04384	0.629999	0.005254	0.020887	1	0.83692
Q99KV1	DnaJ homolog subfamily B member 11	Dnajb11	0.927705	0.735371	0.039137	0.005519	1	0.831538
O89020	Afamin	Afm	0.732094	0.922742	0.004607	0.016608	1	0.827418
Q61702	Inter-alpha-trypsin inhibitor heavy chain H1	Itih1	0.749531	0.900243	0.003662	0.002623	1	0.824887
Q61730	Interleukin-1 receptor accessory protein	Il1rap	0.757866	0.879799	0.009336	0.003604	1	0.818833
Q61129	Complement factor I	Cfi	0.727561	0.909132	0.03009	0.037969	1	0.818347
Q9QXC1	Fetuin-B	Fetub	0.75059	0.87121	8.95E-05	0.002474	1	0.8109
Q01339	Beta-2-glycoprotein 1	Apoh	0.646425	0.973584	0.004914	0.010689	1	0.810005
Q8CG14	Complement C1s-A subcomponent	C1sa	0.627032	0.989454	0.008279	0.006796	1	0.808243
Q03734	Serine protease inhibitor A3M	Serpina3m	0.83729	0.767097	0.003976	0.002533	1	0.802194
Q61646	Haptoglobin	Hp	0.59905	0.998107	0.003728	0.008036	1	0.798579
P29788	Vitronectin	Vtn	0.703535	0.8806	0.005271	0.004495	1	0.792068
P00687	Alpha-amylase 1	Amy1	0.72728	0.849528	0.000263	0.000925	1	0.788404
P39061	Collagen alpha-1(XVIII) chain	Col18a1	0.646738	0.921492	0.014931	0.0198	1	0.784115
Q07456	Protein AMBP	Ambp	0.738933	0.824178	0.010664	0.009883	1	0.781556
Q9DD06	Retinoic acid receptor responder protein 2	Rarres2	0.705175	0.853459	0.021217	0.019423	1	0.779317
Q9Z1R3	Apolipoprotein M	Apom	0.622733	0.882773	0.005584	0.031497	1	0.752753
P21614	Vitamin D-binding protein	Gc	0.484598	1.01567	0.006939	0.009621	1	0.750134
A0A0R4J0X5	Alpha-1-antitrypsin 1-3	Serpina1c	0.756974	0.741501	0.005008	0.008425	1	0.749238

A0A0R4J0E1	Fibrinogen-like protein 1	Fgl1	0.776398	0.68763	0.015769	0.013806	1	0.732014
G3X8T9	Serine protease inhibitor A3N	Serpina3n	0.817154	0.63954	0.001787	0.001223	1	0.728347
Q9JJH1	Ribonuclease 4	Rnase4	0.703282	0.75037	0.002387	0.003945	1	0.726826
Q61247	Alpha-2-antiplasmin	Serpinf2	0.653313	0.786254	0.001811	0.009316	1	0.719784
Q61838	Alpha-2-macroglobulin	A2m	0.538236	0.867365	0.00432	0.002132	1	0.702801
P20918	Plasminogen	Plg	0.634434	0.7614	0.000918	0.00254	1	0.697917
Q61703	Inter-alpha-trypsin inhibitor heavy chain H2	Itih2	0.571916	0.818447	0.002672	0.013441	1	0.695182
P28665	Murinoglobulin-1	Mug1	0.569547	0.820048	0.005395	0.008677	1	0.694798
P16301	Phosphatidylcholine-sterol acyltransferase	Lcat	0.481351	0.895242	0.031305	0.021457	1	0.688297
P06684	Complement C5	C5	0.608022	0.758318	0.004888	0.001704	1	0.68317
Q00897	Alpha-1-antitrypsin 1-4	Serpina1d	0.622752	0.739555	0.00069	0.000837	1	0.681154
Q61704	Inter-alpha-trypsin inhibitor heavy chain H3	Itih3	0.594167	0.765444	0.016065	0.034705	1	0.679806
Q91X70	Complement component 6	C6	0.650345	0.703697	0.002664	0.001472	1	0.677021
Q543J5	Antithrombin-III	Serpinc1	0.552902	0.779737	0.002375	0.021326	1	0.66632
P52430	Serum paraoxonase/arylesterase 1	Pon1	0.796431	0.527249	8.37E-05	0.002997	1	0.66184
Q8K0E8	Fibrinogen beta chain	Fgb	0.505452	0.81193	0.004523	0.003733	1	0.658691
P23953	Carboxylesterase 1C	Ces1c	0.584191	0.718944	0.009152	0.017511	1	0.651568
E9PV24	Fibrinogen alpha chain [Cleaved into: Fibrinopeptide A; Fibrinogen alpha chain]	Fga	0.514974	0.768713	0.001345	0.009826	1	0.641844
A0A0R4J0I1	MCG1051009	Serpina3k	0.59091	0.684522	0.000894	0.007934	1	0.637716

Q8QZR3	Pyrethroid hydrolase Ces2a	Ces2a	0.883747	0.385019	0.004935	0.004238	1	0.634383
Q3UER8	Fibrinogen gamma chain	Fgg	0.493163	0.774318	0.006685	0.01419	1	0.633741
Q546G4	Serum albumin	Alb	0.433894	0.802888	0.006671	0.022761	1	0.618391
P11859	Angiotensinogen	Agt	0.641133	0.584907	0.000715	0.006628	1	0.61302
Q3KQQ2	Major urinary protein 3	Mup3	0.626035	0.596879	0.006649	0.000263	1	0.611457
P47876	Insulin-like growth factor-binding protein 1	Igfbp1	0.580634	0.598306	0.001574	0.006149	1	0.58947
Q63886	UDP-glucuronosyltransferase 1-1	Ugt1a1	0.799529	0.348491	0.000175	0.00379	1	0.57401
Q8VCU1	Carboxylesterase 3B	Ces3b	0.756702	0.390716	0.001796	0.001964	1	0.573709
A2BIN1	Major urinary protein 6	Mup10	0.633616	0.435789	0.000143	0.002796	1	0.534703
Q3UEK9	Alpha-2-HS-glycoprotein	Ahsg	0.362459	0.660603	0.039302	0.007872	1	0.511531
Q80W65	Proprotein convertase subtilisin/kexin type 9	Pcsk9	0.442256	0.575119	0.002658	0.001096	1	0.508688
Q8BH35	Complement component C8 beta chain	C8b	0.466107	0.548211	0.004736	0.003756	1	0.507159
Q9WVF5	Epidermal growth factor receptor	Egfr	0.483555	0.509544	0.003999	0.003647	1	0.49655
Q8BWQ1	UDP-glucuronosyltransferase 2A3	Ugt2a3	0.636476	0.256776	0.00522	0.005175	1	0.446626
Q8K169	UDP-glucuronosyltransferase 2B17	Ugt2b5	0.632705	0.248918	0.005666	0.010876	1	0.440812
B5X0G2	Major urinary protein 17	Mup17	0.621817	0.220358	0.01099	0.03151	1	0.421088
Q8R084	UDP-glucuronosyltransferase	Ugt2b1	0.454855	0.201762	0.003923	0.03144	1	0.328309
A2CEK7	Major urinary protein 12	Mup14	0.345006	0.221362	0.001517	0.001057	1	0.283184
Q00898	Alpha-1-antitrypsin 1-5	Serpina1e	0.145514	0.154529	0.006621	0.007123	1	0.150022

Supplementary Table 1. The primers for quantitative PCR

Mouse		
Gene name	FOR	REV
Gapdh	ACGGCAAATTCAACGGCACAG	AGACTCCACGACATACTCAGCAC
Tnf	AGGGTCTGGGCCATAGAACT	CCACCACGCTCTTCTGTCTA
Il1b	CTCGCAGCAGCACATCAACAAG	CCACGGGAAAGACACAGGTAGC
Il6	ACAAAGCCAGAGTCCTTCAGAGAG	TTGGATGGTCTTGGTCCTTAGCC
Il10	GCTGGACAACATACTGCTAACCG	TCCGATAAGGCTTGGCAACCC
Arg1	CTCCAAGCCAAAGTCCTTAGAG	AGGAGCTGTCATTAGGGACATC
Cd163	CTGGCGGGTGGTGAAAACA	CAGCCGTTACTGCACACTG
Nos2	GCACCACCCTCCTCGTTCAG	TCCACAACTCGCTCCAAGATTCC
Ccl2	ATTGGGATCATCTTGCTGGT	CCTGCTGTTCACAGTTGCC
Ccl3	TCCCAGCCAGGTGTCATTTTCC	CAGTTCCAGGTCAGTGATGTATTCTTG
Ccl4	CTCTGCGTGTCTGCCCTCTC	TGGTCTCATAGTAATCCATCACAAAGC
Ccl5	ACTCCCTGCTGCTTTGCCTAC	TGTATTCTTGAACCCACTTCTTCTCTG
Cxcl2	AAAGTTTGCCTTGACCCTGAA	CTCAGACAGCGAGGCACATC
Cxcl10	GACGGTCCGCTGCAACTG	CTTCCCTATGGCCCTCATTCT
Col1a1	ACAGGCGAACAAGGTGACAGAG	GCCAGGAGAACCAGCAGAGC
Col1a2	GGGCAACAGCAGGTTCACCTA	AATGTCCAGAGGTGCAATGTCAAG
Col3a1	CAGGCCAGTGGCAATGTAAAGA	CTCATTGCCTTGCGTGTTTGATA
Col4a1	AGGAGAGAAGGGTGAACAAGGTG	CCAGGAGTGCCAGGTAAGCC
Col5a1	CTTCGCCGCTACTCCTGTTC	CCCTGAGGGCAAATTGTGAAAA
Lox	GGTTACTTCCAGTACGGTCTCC	GCAGCGCATCTCAGGTTGT
Timp1	TCTGGCATCTGGCATCCTCTTG	AACGCTGGTATAAGGTGGTCTCG
Acta2	TCAGGGAGTAATGGTTGGAATGGG	CAGTTGGTGATGATGCCGTGTTC
Serpina1c	TCGCCCTGGCAAATTACATTC	GCTGCAATGGTGCACATCAA
Serpina1d	ACCCAGTTCTGGGACAGCAA	TCCCTGTATAGTCTGAGGGCAAAG
Serpina1e	CCTGCTAAACAGGCGCAGA	GTCAGCACGGCCTTATGCAC
HIf1a	TACCACACTGAGGTTGGTTACTGT	AACTAGCCGAGGAAGAACTATGAACA
Hnf4a	CACGCGGAGGTCAAGCTAC	CCCAGAGATGGGAGAGGTGAT
Pr3tn	GAACTGAACGTCACGGTGGTC	AAGAATGCCATTGCAGATCAAG
Human		
Gene name	FOR	REV

GAPDH	ATCACCATCTTCCAGGAGCG	GGCCTCACCCCATTTGATGT
IL-32γ	AGGCCCGAATGGTAATGCT	CCACAGTGTCCTCAGTGTCACA
SERPINA1	AGAGGGCCAGCTAAGTGGTA	GAGGCTGCGAAAGGAGTCAT
HIF1a	CACCACAGGACAGTACAGGAT	CGTGCTGAATAATACCACTCACA
HNF4a	CAGGCTCAAGAAATGCTTC	GGCTGCTGTCCTCATAGCTT

Supplementary Table 2. Lists of Antibodies

Antibody		
Protein	Company	Product Code
IL-32	YbdY	PAB102
ACTIN	Merk	MAB1501
GAPDH	Merk	AB2302
PR3	Santa Cruz	sc-74534
A1AT	ABclonal	A1015
HIF1α	Santa Cruz	sc-13515
HNF4α	Santa Cruz	sc-374229
HNF4a	Santa Cruz	sc-374229 X
APC anti-mouse CD45	BioLegend	103112
APC/Cyanine7 anti-mouse F4/80	BioLegend	123118
PE/Cyanine7 anti-mouse Tim-4	BioLegend	130010
Pharmingen™ FITC Rat Anti-CD11b	BD Biosciences	553310
PE anti-mouse CD192 (CCR2)	BioLegend	150610
JNK1/2/3	ABclonal	A4867
p-JNK	ABclonal	AP0276
ERK	Santa Cruz	sc-514302
p-ERK	Santa Cruz	sc-7383
IκB-α	ABclonal	A19714
p-IκB-α	Cell signaling	2859
P65	ABclonal	A11201
p-P65	Cell signaling	3033S
CLEC4F	R&D Systems	AF2784
PR3	ABclonal	A19748
F4/80	Thermo Fisher Scientific	13-4801-82

Chicken anti-Rabbit IgG (H+L) Cross-Adsorbed Secondary Antibody, Alexa Fluor™ 488	Invitrogen	A-21441
Goat IgG (H+L) PE-conjugated Antibody	R&D Systems	F0107

References

1 Casimiro, I., Stull, N. D., Tersey, S. A. & Mirmira, R. G. Phenotypic sexual dimorphism in response to dietary fat manipulation in C57BL/6J mice. *J Diabetes Complications* **35**, 107795 (2021). https://doi.org:10.1016/j.jdiacomp.2020.107795

2 Oraha, J., Enriquez, R. F., Herzog, H. & Lee, N. J. Sex-specific changes in metabolism during the transition from chow to high-fat diet feeding are abolished in response to dieting in C57BL/6J mice. *Int J Obes (Lond)* **46**, 1749-1758 (2022). https://doi.org:10.1038/s41366-022-01174-4

3 Kim, H. S. *et al.* Reversion of in vivo fibrogenesis by novel chromone scaffolds. *EBioMedicine* **39**, 484-496 (2019). https://doi.org:10.1016/j.ebiom.2018.12.017
